# Effect of l‐oxiracetam and oxiracetam on memory and cognitive impairment in mild‐to‐moderate traumatic brain injury patients: Study protocol for a randomized controlled trial

**DOI:** 10.1002/agm2.12335

**Published:** 2024-06-14

**Authors:** Tao Liu, Mingqi Liu, Meng Nie, Zhihao Zhao, Xuanhui Liu, Yu Qian, Yunhu Yu, Zhuang Sha, Chenrui Wu, Jiangyuan Yuan, Weiwei Jiang, Chuanxiang Lv, Liang Mi, Yu Tian, Jianning Zhang, Rongcai Jiang

**Affiliations:** ^1^ Department of Neurosurgery Tianjin Neurological Institute, State Key Laboratory of Experimental Hematology, Key Laboratory of Post‐Neuroinjury Neurorepair and Regeneration in Central Nervous System Tianjin & Ministry of Education, Tianjin Medical University General Hospital Tianjin China; ^2^ Department of Rehabilitation Medicine Zhejiang Provincial People's Hospital Hangzhou China; ^3^ Department of Clinical Research Center for Neurological Disease The People's Hospital of HongHuaGang District of ZunYi Zunyi China; ^4^ Department of Neurosurgery The First Hospital of Jilin University Changchun China; ^5^ State Key Laboratory of Experimental Hematology Tianjin Medical University General Hospital Tianjin China

**Keywords:** cognition, memory, oxiracetam, randomized controlled trial, traumatic brain injury

## Abstract

**Objectives:**

Patients with traumatic brain injury (TBI) often suffer memory and cognitive impairments, and oxiracetam‐like drugs are considered to have a positive impact on these symptoms potentially. However, the efficacy and safety of l‐oxiracetam and oxiracetam in TBI patients have not been sufficiently investigated.

**Methods:**

The study adopts a multicenter, randomized, double‐blind, parallel‐group, phase 3 clinical trial design in 74 centers across 51 hospitals in China. A total of 590 TBI patients meeting criteria will be randomly allocated into three groups in a 2:2:1 ratio: l‐oxiracetam group, oxiracetam group, and placebo group. The treatment period is 14 days, with a follow‐up period of 90 days. The primary outcome measure is the change in the Loewenstein Occupational Therapy Cognitive Assessment score at 90 days after treatment. Secondary outcomes include changes in other cognitive assessments, neurological function, activities of daily living, and safety assessments.

**Discussion:**

There is no robust evidence to suggest that l‐oxiracetam and oxiracetam can enhance memory and cognitive function in patients with mild to moderate TBI. This study has the potential to answer this crucial clinical question.

**Trial registration:**

chinadrugtrials.org.cn, identifier CTR20192539; ClinicalTrials.gov, identifier NCT04205565.

## Graphical abstract

Study protocol of a multicenter, randomized, double‐blind, parallel‐group clinical trial on l‐oxiracetam and oxiracetam in patients with traumatic brain injury.

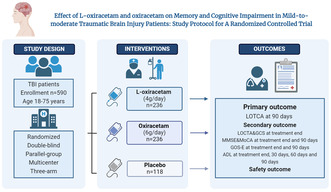

## 1 INTRODUCTION

Traumatic brain injury (TBI) is a significant global health concern,^1^ affecting over 50 million people annually and imposing a considerable burden on healthcare systems and economies.^2^ Its incidence has increased dramatically over the past 30 years, with projections indicating that approximately half of the world's population will experience TBI, impacting individuals, families, and society incalculably.^2^, ^3^ Mild TBI is the most prevalent type, with an incidence rate of 224 cases per 100,000, surpassing the rates of moderate and severe TBI by 10 and 17 times, respectively.^4^, ^5^ Advances in medical technology and healthcare management have led to decreased mortality rates among TBI patients, particularly in moderate to severe cases, shifting focus to understanding the long‐term neurobehavioral outcomes post‐injury.^6^, ^7^ Research suggested that 10% of mild TBI cases manifest enduring functional impairments, while this percentage can exceed 60% for moderate to severe TBI cases.^6^

The limb dysfunction, behavioral and emotional disturbances, and cognitive deficits resulting from TBI impose substantial burdens on both families and society. Among these, cognitive impairment is the most prevalent and enduring, significantly hindering the enhancement of the quality of daily life and social activities for affected individuals.^8^, ^9^ Nevertheless, the mechanisms underlying cognitive dysfunction are intricate and may be associated with structural damage to relevant brain tissues, abnormalities in neurotransmitters and their associated receptors, among other factors.^10^, ^11^, ^12^ Despite the relatively high incidence of cognitive impairment following TBI, it has not garnered as much attention in clinical practice when compared to conditions such as Alzheimer's disease or vascular dementia.^13^, ^14^ The identification of suitable pharmaceutical interventions for cognitive impairment post‐TBI remains a contentious focal point and a research hotspot.

Oxiracetam, a cyclic derivative of gamma‐aminobutyric acid (GABA), belonging to the pyrrolidone nootropics, has been clinically employed to enhance memory and cognitive function.^15^ It exists in two enantiomers: (S)‐oxiracetam and (R)‐oxiracetam.^16^ Early studies suggested that (S)‐oxiracetam has advantages over (R)‐oxiracetam in enhancing cognition, reducing pathological damage, and increasing cerebral blood flow.^17^, ^18^, ^19^ However, there is currently a lack of large‐scale randomized controlled trials and evidence‐based medicine regarding the treatment of post‐TBI memory and cognitive dysfunction with oxiracetam and its enantiomers. Existing therapeutic insights are derived from studies with small sample sizes or research on cognitive impairments from other conditions, such as Alzheimer's disease, vascular dementia, etc.^19^, ^20^, ^21^

To address these deficiencies, we are initiating a randomized, double‐blind, multicenter, phase III large‐scale clinical trial to investigate and compare the efficacy and safety of (S)‐oxiracetam (Trade Name: l‐oxiracetam) and oxiracetam in improving memory and cognitive impairments in patients with mild to moderate TBI. Based on previous research, we anticipate l‐oxiracetam to show superior therapeutic effects compared to oxiracetam. We expect this study to provide robust evidence regarding the efficacy and safety of l‐oxiracetam and oxiracetam in ameliorating memory and cognitive impairments in TBI patients.

## 2 METHODS

### 2.1 Design

This multicenter study is a randomized, double‐blind, parallel, three‐arm, phase III clinical trial. The design and implementation of this trial strictly adhere to the Helsinki Declaration and have obtained approval from the ethics committees of each research center. This trial was approved by the China National Medical Products Administration (Approval Number, 2016L03521) and registered on chinadrugtrials.org.cn (identifier, CTR20192539) and ClinicalTrials.gov (identifier, NCT04205565). The rights and safety of the subjects in this trial are prioritized over the benefits to science and society.

The trial is planned to be conducted in 74 centers across 51 hospitals in China (see Appendix S1), and subjects will not be enrolled in the study until written informed consent (see Appendix S2) is obtained from the subjects and/or their guardians/legal representatives. All qualified medical centers undergo rigorous training before the start of the study and adhere to standardized treatment for enrolled patients.

**FIGURE 1:**
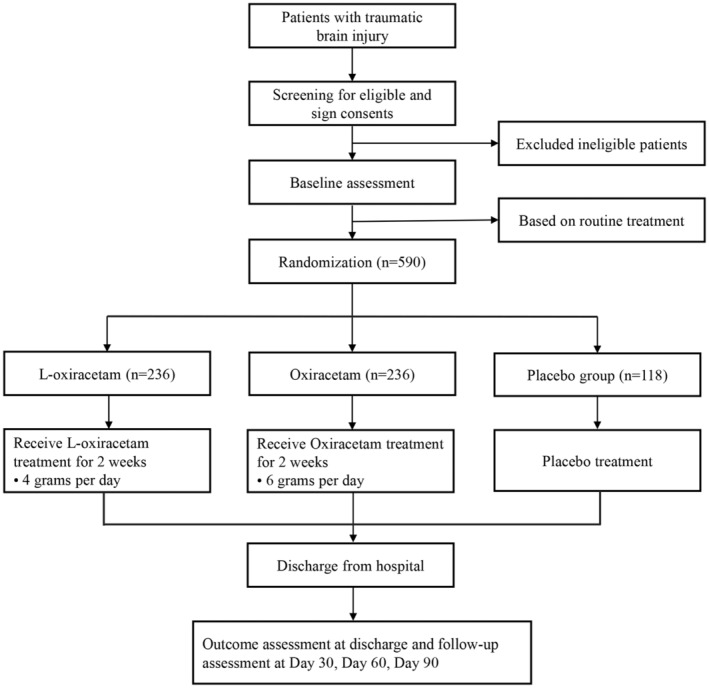
The flowchart of the phase III clinical study of l‐oxiracetam.

Eligible TBI patients will be randomly assigned to three groups: l‐oxiracetam group, oxiracetam group, and placebo group in a ratio of 2:2:1. The efficacy and safety of improving memory and cognitive impairment in TBI patients will be observed over a treatment period of 14 days, with a follow‐up period of 90 days. The flowchart is shown in Figure 1, and the assessment time points are detailed in Table 1.

**TABLE 1:** Time schedule.

Visit window	Medication period		Follow‐up^a^ (±3 days)		
	Screening	Treatment period (14 days + 1 day)	Day 30	Day 60	Day 90
General procedures					
Informed consent	X				
Medical history	X				
General data	X				
Acute phase treatment	X				
Inclusion/Exclusion criteria	X				
Concomitant diseases and medications	X				
Screening indicators					
Pregnancy test (for women of childbearing age)	X				X
CT or MRI	X^b^				X
Safety					
Vital signs	X	X			X
Laboratory tests^c^	X^b^	X			X
12‐lead electrocardiogram	X^b^	X			X
Efficacy					
MMSE	X	X			X
GCS	X	X			
GOS‐E		X			X
MoCA	X	X			X
LOTCA	X	X			X
ADL‐BI		X	X	X	X
Other tasks					
Allocation of drug numbers	X				
Dispensing experimental drugs	X				
Retrieval of remaining drugs		X			
Recording concomitant medications		X	X	X	X
Adverse event recording		X	X	X	X
Efficacy assessment		X			X
Summary of trial completion status					X

Abbreviations: ADL‐BI, Barthel Index of Activities of Daily Living; CT, computed tomography; GCS, Glasgow Coma Scale; GOS‐E, Glasgow Outcome Scale‐Extended; LOCTA, Loewenstein Occupational Therapy Cognitive Assessment; MMSE, Mini‐Mental State Examination; MoCA, Montreal Cognitive Assessment; MRI, magnetic resonance imaging.

^a^ If a participant experiences any discomfort or adverse effects, an unscheduled visit should be arranged.

^b^ For those who have undergone post‐injury examinations, the results should be retained, and repetition of the tests may be waived after obtaining informed consent.

^c^ All materials are archived for for secondary readouts; laboratory tests include blood routine (WBC, RBC, HGB, PLT), urine routine (PRO, GLU, LEU, ERY), liver function (AST, ALT, TBIL, γ‐GT, ALP), kidney function (Scr, GFR), coagulation function tests (PT, APTT, TT, FIB), creatine kinase (CK), and triglycerides (TG).

### 2.2 Study population

Participants will be recruited from 74 centers across 51 hospitals in China for a duration of 2 years. The members of the research team are listed in Appendix S1, and all participants must meet all the inclusion and exclusion criteria listed in Table 2. The trial plans to enroll a total of 590 subjects, with 236 in the l‐oxiracetam group, 236 in the oxiracetam group, and 118 in the placebo group.

**TABLE 2:** Inclusion and exclusion criteria.

Inclusion criteria	Exclusion criteria
Age 18–75 years; Head injury meeting all of the following conditions: (1) clear evidence of head trauma in the current diagnosis, including closed head injury or head injury with cerebrospinal fluid leakage and/or ear or nasal leakage and/or intracranial air accumulation; (2) confirmed by MRI or CT to have intracranial bleeding above the cerebellar tentorium (including cerebral contusion, subarachnoid hemorrhage, extradural hematoma, subdural hematoma, intracerebral hematoma, etc.), with or without transient loss of consciousness; (3) classified as mild to moderate head injury (GCS: 9–15); (4) Stable condition within 72 h after head injury, undergoing conservative treatment, not undergoing craniotomy (may have intracranial pressure monitoring without general anesthesia or basal anesthesia); MMSE score below normal, with diagnostic cutoff values depending on different educational levels: illiterate (no education) ≤19 points, elementary school level ≤ 22 points, junior high school and above level ≤ 26 points; Consent from the guardian and/or patient to participate in this clinical trial and signing of the informed consent form.	Known or suspected allergy to the experimental drug or its components; Use of prohibited drugs or other cognitive‐enhancing drugs listed in the protocol after injury; History of severe head trauma, cerebrovascular accidents, or structural brain lesions; Conditions such as speech/hearing impairment that prevent completion of cognitive function assessment; Occurrence of secondary brain injury after the current head injury; Need for craniotomy or external ventricular drainage; Concurrent serious injuries to other major organs or serious complications that may affect the subject's life; Patients with active epilepsy within the past year; Severe liver or kidney disease with abnormal liver or kidney function tests (ALT, AST ≥3 times the upper limit of normal, Scr > upper limit of normal); Concurrent severe heart disease, lung disease, blood and hematopoietic system diseases, gastrointestinal diseases, or other severe or progressive systemic diseases; History or current diagnosis of malignant tumors (excluding cured stage IB or lower cervical cancer, non‐invasive basal cell or squamous cell skin cancer; exclusion of breast cancer with CR > 10 years, malignant melanoma with CR > 10 years, and other malignant tumors with CR >5 years); Presence of neurological or psychiatric disorders that prevent or unwillingness to cooperate; Pregnant, lactating women, or those with recent plans for childbirth; Investigator deems unsuitable for participation in the clinical trial; Participation in another clinical trial and use of experimental drugs in the last 3 months before the trial.

Abbreviations: ALT, alanine aminotransferase; AST, aspartate aminotransferase; CR, complete remission; CT, computed tomography; GCS, Glasgow Coma Scale; MMSE, Mini‐Mental State Examination; MRI, magnetic resonance imaging; Scr, serum creatinine.

### 2.3 Blinding

This trial employs a placebo as controls and adopts a double‐blind design. Placebo medications for l‐oxiracetam and oxiracetam were prepared by the sponsor to achieve blinding. Simulated agents are uniformly packaged, ensuring that drug categories cannot be distinguished by appearance and meeting preparation requirements.

Additionally, each clinical trial unit designated specific research nurses for drug dispensing and administration, ensuring they did not disclose drug allocation to participants, were not involved in trial assessments, and were solely responsible for drug preparation and administration. Unblinding procedures could be initiated through the Interactive Web Response System (IWRS) in case of serious adverse events, deterioration, or disease progression in participants.

The double‐blind design involves first‐level blinding, where the blind codes correspond to the treatment groups (l‐oxiracetam, oxiracetam, or placebo) assigned to each case number. The randomization code list was created by the statistical unit, and the blind codes were individually sealed in duplicate and stored separately at the leading unit and the sponsor's site.

### 2.4 Randomization

In this trial, a block randomization method will be used with a ratio of 2:2:1, where successfully screened subjects will be randomly assigned to three groups. The study employs a centralized randomization procedure, with each center competing for participant enrollment. The Department of Biostatistics at the School of Public Health, Nanjing Medical University, provides centralized randomization system. Following the confirmation of each eligible subject by the center's investigator, researchers at each center will log into the system, input screening data, obtain a random number and drug code, and dispense the investigational drug accordingly.

### 2.5 Treatment and intervention

Phase I clinical trials showed that l‐oxiracetam was well tolerated and safe when administered multiple times within the dose range of 0.6 to 8.0 g. In Phase II exploratory clinical trials, a 1:1:1:1 ratio was used to explore the efficacy and safety of l‐oxiracetam injection (at doses of 3 and 4 g/day), oxiracetam infusion, and placebo. A total of 200 subjects were enrolled.

Preliminary statistical analysis of the Phase II clinical study (Per Protocol Set) showed that after 3 months of treatment cessation, the change in the total score of the Loewenstein Occupational Therapy Cognitive Assessment (LOTCA) scale from the baseline in the high‐dose group (4 g/day), low‐dose group (3 g/day), oxiracetam injection group, and placebo group were 26.0 ± 11.8, 22.2 ± 10.3, 22.8 ± 10.0, and 20.0 ± 11.4, respectively. The high‐dose group (4 g/day) showed statistical significance compared to the placebo group (*p* < 0.05), while the low‐dose group (3 g/day) did not show statistical significance compared to the placebo group but exhibited an increasing trend. No serious adverse events related to the investigational drug were reported in any of the subjects, indicating overall good tolerability and safety.

Based on the efficacy and safety results from previous studies, this trial will adopt a double‐blind, double‐dummy administration method, with all participants receiving l‐oxiracetam (4 g/day), oxiracetam (6 g/day), or placebo treatment for 14 days.

Prohibited treatments and medications during the trial are listed in Appendix S3.

### 2.6 Drug provision

l‐oxiracetam: Injectable l‐oxiracetam, specification: 1 g per vial, manufactured by Shenghe (China) Biopharma Co., Ltd.

Oxiracetam: Injectable oxiracetam, specification: 1 g per vial, manufactured by Shiyao Group Ouyi Pharmaceutical Co., Ltd.

Placebo: Injectable l‐oxiracetam or oxiracetam placebo with identical color, smell, and appearance but without active ingredients, manufactured by Shenghe (China) Biopharma Co., Ltd.

All investigational drugs will be provided by Nanjing Yoko Pharmaceutical Co., Ltd. in accordance with blinding requirements and meet quality standards.

### 2.7 Outcome measures

#### 2.7.1 Efficacy outcomes

The primary outcome measure is the change in scores on the LOTCA at 90 days post‐treatment compared to the baseline.

The secondary outcome measures include: (1) changes in scores on the LOTCA and Glasgow Coma Scale (GCS) at the end of treatment compared to the baseline; (2) changes in scores on the MMSE and Montreal Cognitive Assessment (MoCA) at the end of treatment and 90 days post‐treatment compared to the baseline; (3) the percentage of subjects at each level of the Glasgow Outcome Scale‐Extended (GOS‐E) at the end of treatment and 90 days post‐treatment; and (4) Barthel Index of Activities of Daily Living (ADL‐BI) at the end of treatment, 30, 60, and 90 days.

Detailed assessment scales and operating instructions are available in Appendix S4.

#### 2.7.2 Safety outcomes

1. Vital signs (temperature, pulse, respiration, and blood pressure);
2. The laboratory assessment includes complete blood count, blood biochemistry, coagulation function, liver and kidney function, triglycerides, creatine kinase, and urine examination;
3. 12‐lead electrocardiogram (ECG);
4. Adverse events: Refers to any unfavorable medical occurrence in a participant, which may manifest as symptoms, signs, diseases, or abnormalities in laboratory tests, but not necessarily causally related to the investigational drug.

### 2.8 Follow‐up

After completing the 14‐day treatment, follow‐up assessments will be conducted at 30, 60, and 90 days post‐treatment for all participants. The specific evaluation content can be referred to Table 1.

### 2.9 Sample size calculation

Before conducting this study, we conducted a pilot trial: The change values of the total scores on the LOTCA scale at 90 days post‐drug cessation compared to the baseline for l‐oxiracetam and oxiracetam were 26.0 ± 11.8 and 22.8 ± 10.0, respectively. The combined standard deviation for both groups was 11.0, with *α* set at 0.05 and 1 − *β* at 0.8. Employing an optimal design with a sample ratio of 1:1, PASS software calculated a sample size of 190 cases for each group.

The change values of the total scores on the LOTCA scale at 90 days post‐drug cessation compared to the baseline for the l‐oxiracetam group and the placebo group were 26.0 ± 11.8 and 20.0 ± 11.4, respectively. The combined standard deviation for both groups was 11.6, with *α* set at 0.05 and 1 − *β* at 0.8. Employing an optimal design with a sample ratio of 2:1, PASS software calculated a sample size of 204 cases for the l‐oxiracetam group and 102 cases for the placebo group.

Considering potential dropouts, we increased the sample size by 15%. Therefore, the sample sizes for the l‐oxiracetam group, oxiracetam group, and placebo group were set at 236, 236, and 118, respectively.

### 2.10 Statistical analyses

SAS 9.4 was used for data analysis independently conducted by a biostatistician unaware of the allocation. Continuous variables will be presented as mean ± standard deviation (SD) for normal distribution and as median (range) for abnormal distribution. Categorical variables will be expressed by numbers and percentages.

All hypothesis tests were two‐sided with *α* = 0.05. A *p*‐value < 0.05 indicated statistical significance. Analysis followed the Intention‐To‐Treat (ITT) principle, including all randomized cases receiving at least one drug dose. For cases with incomplete treatment process, the last observed data were carried forward to the trial's final results.

When comparing the change values from the baseline for each group, an analysis of covariance (ANCOVA) model was used, adjusting for the baseline level (i.e., each outcome), age, sex, education, and injury severity. Cohen's d with 95% confidence intervals will be used to calculate the effect sizes. Adverse event data, coded according to MedDRA 26.0, were processed in the statistical analysis.

### 2.11 Data management and quality control

This study employs an electronic case report form (eCRF) for data collection and management, ensuring traceability with complete modification trails. Data management follows good clinical practice (GCP) specifications, with audits conducted by data managers based on the data verification plan (DVP). Audits focus on aspects like missing data, logical issues, timeframes, inclusion/exclusion criteria, medication compliance, and consistency checks. After the initial audit, a meeting is held to review the data and allocate analysis datasets. Following this, the database is locked, and materials are handed over to statisticians for analysis.

## 3 DISCUSSION

This multicenter study is a randomized, double‐blind, parallel‐group, three‐arm, phase III clinical trial. The primary objective is to investigate the efficacy and safety of l‐oxiracetam and oxiracetam in improving memory and cognitive impairment in patients with mild to moderate TBI. Based on previous findings, we aim to validate our hypothesis that l‐oxiracetam is superior to oxiracetam and placebo in ameliorating memory and cognitive impairment in patients with mild to moderate TBI. We anticipate that the results will provide crucial empirical data for the application of l‐oxiracetam and oxiracetam in patients with mild to moderate TBI, thereby positively influencing clinical practices and treatment strategies in related fields.

Oxiracetam, a pyrrolidinone derivative, is a nootropic drug known to enhance cognitive function, including executive functions, memory, creativity, and motivation. The most widely accepted hypothesis is that these types of drugs can activate central cholinergic neurons directly or indirectly, leading to an increase in the synthesis or release of acetylcholine, enhancement of cholinergic receptor sensitivity, or modulation of the activity of acetylcholinesterase, subsequently modulating cognitive function.^22^, ^23^, ^24^, ^25^, ^26^ Clinical trials have shown that oxiracetam can alleviate physical and psychiatric symptoms in patients with primary degenerative and multi‐infarct dementia, and to some extent, improve cognitive impairments in dementia patients.^27^, ^28^, ^29^

Moreover, early studies have reported different pharmacological effects of the two enantiomers of oxiracetam, with (S)‐oxiracetam often showing superior activity and benefits compared to (R)‐oxiracetam.^17^ Pharmacokinetic studies in animals have shown significantly higher absorption rates for (S)‐oxiracetam compared to (R)‐oxiracetam after oral administration, with minimal differences in distribution, metabolism, or excretion processes, and only about a 2% interconversion between the enantiomers. According to classical pharmacological theory, a higher absorption rate corresponds to a higher concentration and stronger effects. This suggests that the varying effects of (S)‐oxiracetam and (R)‐oxiracetam may be partially attributed to their distinct absorption rates.^30^, ^31^

In recent years, other potential mechanisms for improving cognitive impairment by oxiracetam have been discovered, sparking interest among researchers. Studies have shown that in the chronic cerebral hypoperfusion (CCH) model of adult rats, oxiracetam can ameliorate cognitive impairments induced by CCH, preventing defects in neural plasticity, white matter damage, and synaptic ultrastructure.^32^ Additionally, it has been confirmed that (S)‐oxiracetam is effective in alleviating cognitive dysfunction induced by CCH, possibly by inhibiting abnormal adenosine triphosphate (ATP) metabolism and increasing the levels of small molecules such as glutamate and glutamine in the cortical region.^18^

Furthermore, oxiracetam shows promise in improving blood–brain barrier disruption after ischemic stroke and inducing a neuroprotective microglial phenotype after hypoxic–ischemic brain injury.^33^, ^34^ However, research on the effects of oxiracetam and its enantiomers after TBI is limited. Studies have shown that intraperitoneal injection of oxiracetam can improve the performance of TBI mice in water maze and Y maze experiments and reduce inflammation and apoptosis markers in brain tissue.^35^ Similarly, intragastric administration with oxiracetam in TBI rats has yielded similar behavioral results.^36^ Overall, oxiracetam is an effective class of nootropic drugs with established efficacy and good patient tolerance. Adverse reactions, mainly affecting the digestive system, typically resolve upon discontinuation, contributing to its widespread use.^37^, ^38^

Despite the negative impact of TBI on patients' cognitive function, there is a lack of specific and effective drug treatments to improve cognitive function post these injuries. This gap is particularly notable in the clinical realm, especially when focusing on patients with mild to moderate TBI—an area that is relatively common but lacks robust research. Oxiracetam‐like drugs have sparked research interest as potential agents for improving cognitive function. However, there is currently limited understanding of the relative effectiveness and safety of l‐oxiracetam and oxiracetam in patients with TBI. In the current study, we posed a question: Is there a difference in the efficacy of l‐oxiracetam compared to oxiracetam in treating memory and cognitive impairments in patients with mild to moderate TBI? While some potential differences were noted in previous research, our primary objective in this trial is to obtain a more comprehensive insight into their practical effectiveness in TBI patients.

In addition to the planned analysis, we are making additional efforts to explore the long‐term effects of the interventions and delve into the mechanisms. We are considering extending the follow‐up period to 2 years to thoroughly assess the lasting impacts of l‐oxiracetam and oxiracetam on cognitive function in TBI patients. This extended timeframe will offer valuable insights into treatment sustainability and any potential delayed effects. Conducting subgroup analyses to explore whether the efficacy and safety of l‐oxiracetam and oxiracetam vary across different patient subpopulations (e.g., age, gender, severity of TBI) is part of our initiative, helping to identify patient characteristics that predict treatment response and inform personalized treatment approaches. Additionally, we intend to initiate mechanistic studies while obtaining preliminary results from clinical trials to investigate the underlying mechanisms of action of l‐oxiracetam and oxiracetam in affecting cognitive impairment post‐TBI. These studies could involve neuroimaging techniques, molecular assays, or animal models to elucidate the neurobiological pathways involved in the drugs' effects on cognitive function.

Lastly, this study has some potential limitations, including a predominantly Chinese sample, limiting generalizability to patients in different disease states. Due to ethnic differences, pharmacokinetic parameters may vary. To address this, future research could involve collaboration with international centers to recruit a more diverse participant pool, allowing for a broader understanding of treatment effects across various ethnicities. Additionally, sample collection timing may constrain the acquisition of certain parameters, impacting a comprehensive understanding of drug metabolism. Finally, the assessment of dose ratios is based on specific standards, which may be overly stringent across the entire dosage range, suggesting the exploration of alternative dosing strategies in future studies.

## 4 SUMMARY AND CONCLUSIONS

This study designed a multicenter, randomized, double‐blind, parallel, phase 3 clinical trial aimed to investigating and comparing the efficacy and safety of l‐oxiracetam and oxiracetam in treating memory and cognitive impairments in patients with mild to moderate TBI. Through this research, we aim to uncover the therapeutic potential of l‐oxiracetam and oxiracetam in TBI patients, providing a more scientifically grounded and reliable treatment option for clinical practice.

## AUTHOR CONTRIBUTIONS

RJ and JZ contributed to the study's concept and design. TL, ML, and MN drafted the article. ZZ, XL, YQ, YY, ZS, CW, and JY critically revised the article for important intellectual content. WJ, CL, LM, and YT provided supervision for the study. All authors approved the submitted version.

## CONFLICT OF INTEREST STATEMENT

The authors declare that the research was conducted in the absence of any commercial or financial relationships that could be construed as a potential conflict of interest.

## ETHICS STATEMENT

This trial was approved by the China National Medical Products Administration (Approval Number, 2016L03521) and registered on chinadrugtrials.org.cn (identifier, CTR20192539) and ClinicalTrials.gov (identifier, NCT04205565). The trial involving humans was approved by Ethics Committee of Tianjin Medical University General Hospital (IRB2019‐202‐02). The trial was conducted in accordance with the local legislation and institutional requirements. The participants provided their written informed consent to participate in this study. The findings from this research will be presented through submission to a peer‐reviewed scientific journal. The clinical evidence and insights gained from the trial will be shared with participants and the public through conferences and publications.

## Supporting information


Appendix S1.

Appendix S2.

Appendix S3.

Appendix S4.
