# Supplementary material for: Effect of l‐oxiracetam and oxiracetam on memory and cognitive impairment in mild‐to‐moderate traumatic brain injury patients: Study protocol for a randomized controlled trial
Source: Aging Med (Milton). 2024 Jun 14;7(3):341–9. doi: 10.1002/agm2.12335 (PMC11222749; doi:10.1002/agm2.12335)
Supplement: Supplementary file 1 — Appendix S1. [file AGM2-7-341-s002.docx]

**Appendix 1. Participating hospital**

| **No.** | **Institution Name** | **Province - City** |
| --- | --- | --- |
| 1 | Tianjin Medical University General Hospital | Tianjin |
| 2 | Beijing Chao-Yang Hospital, Capital Medical University | Beijing |
| 3 | Inner Mongolia People's Hospital | Inner Mongolia - Hohhot |
| 4 | Air Force Hospital of Northern Theater of PLA | Liaoning - Shenyang |
| 5 | The First People's Hospital of Shenyang | Liaoning - Shenyang |
| 6 | Tonghua Central Hospital | Jilin - Tonghua |
| 7 | Affiliated Hospital of Hebei University | Hebei - Baoding |
| 8 | Shanxi Provincial People's Hospital | Shanxi - Taiyuan |
| 9 | The First Affiliated Hospital of Henan University of Science and Technology | Henan - Luoyang |
| 10 | The First Hospital of Ningbo | Zhejiang - Ningbo |
| 11 | The Affiliated Hospital of Xuzhou Medical University | Jiangsu - Xuzhou |
| 12 | The First People's Hospital of Lianyungang | Jiangsu - Lianyungang |
| 13 | Suzhou Kowloon Hospital | Jiangsu - Suzhou |
| 14 | The Affiliated Huaian No.1 People’s Hospital of Nanjing Medical University | Jiangsu - Huai'an |
| 15 | Taizhou People's Hospital | Jiangsu - Taizhou |
| 16 | Northern Jiangsu People's Hospital | Jiangsu - Yangzhou |
| 17 | Affiliated Hospital of Nantong University | Jiangsu - Nantong |
| 18 | Affiliated Hospital of Jiangsu University | Jiangsu - Zhenjiang |
| 19 | The First People's Hospital of Changzhou | Jiangsu - Changzhou |
| 20 | Xiangya Hospital Central South University | Hunan - Changsha |
| 21 | Zhangzhou Municipal Hospital of Fujian Province | Fujian - Zhangzhou |
| 22 | The Second Affiliated Hospital of Guangzhou Medical University | Guangdong - Guangzhou |
| 23 | Guangzhou First People's Hospital | Guangdong - Guangzhou |
| 24 | Huizhou Central People's Hospital | Guangdong - Huizhou |
| 25 | Yichun People's Hospital | Jiangxi - Yichun |
| 26 | The Second Affiliated Hospital of Nanchang University | Jiangxi - Nanchang |
| 27 | The First Affiliated Hospital of Gannan Medical University | Jiangxi - Ganzhou |
| 28 | Haikou People's Hospital | Hainan - Haikou |
| 29 | The First People's Hospital of Nanning | Guangxi - Nanning |
| 30 | The Second Nanning People's Hospital | Guangxi - Nanning |
| 31 | Liuzhou Workers' Hospital | Guangxi - Liuzhou |
| 32 | Liuzhou People's Hospital | Guangxi - Liuzhou |
| 33 | Yan'an Hospital of Kunming City | Yunnan - Kunming |
| 34 | The People's Hospital of Yuechi County | Sichuan - Guang'an |
| 35 | The Second Affiliated Hospital of Shantou University Medical College | Guangdong - Shantou |
| 36 | Jieyang People's Hospital | Guangdong - Jieyang |
| 37 | Xianyang Hospital of Yan'an University | Shaanxi - Xianyang |
| 38 | Pu'er People's Hospital | Yunnan - Puer |
| 39 | The First Affiliated Hospital of Nanchang University | Jiangxi - Nanchang |
| 40 | Yangquan Coal Industry (Group) General Hospital | Shanxi - Yangquan |
| 41 | Zhejiang Provincial People's Hospital | Zhejiang - Hangzhou |
| 42 | Taizhou First People's Hospital | Zhejiang - Taizhou |
| 43 | Chengdu Xinhua Hospital | Sichuan - Chengdu |
| 44 | The People's Hospital of Dazhu County | Sichuan - Dazhou |
| 45 | The Affiliated Hospital of Southwest Medical University | Sichuan - Luzhou |
| 46 | The Fifth Affiliated Hospital of Guangzhou Medical University | Guangdong - Guangzhou |
| 47 | The Third Affiliated Hospital of Guangzhou Medical University | Guangdong - Guangzhou |
| 48 | Affiliated Hospital of Zunyi Medical University | Guizhou - Zunyi |
| 49 | Zhumadian Central Hospital | Henan - Zhumadian |
| 50 | Shiyan Renmin Hospital | Hubei - Shiyan |
| 51 | The Second Hospital of Tianjin Medical University | Tianjin |
