# Supplementary material for: Effect of l‐oxiracetam and oxiracetam on memory and cognitive impairment in mild‐to‐moderate traumatic brain injury patients: Study protocol for a randomized controlled trial
Source: Aging Med (Milton). 2024 Jun 14;7(3):341–9. doi: 10.1002/agm2.12335 (PMC11222749; doi:10.1002/agm2.12335)
Supplement: Supplementary file 2 — Appendix S2. [file AGM2-7-341-s003.docx]

# Appendix 2

# Informed Consent Form · Notification Page

| Protocol Number: NJYK-L-ORCT-III |  |
| --- | --- |
| Research Center Name: |  |
| Research Center Address: |  |
| Patient Name: | Patient Screening Number: |

We are about to conduct a randomized, double-blind, parallel control, multicenter, Phase III clinical study on the use of L-oxiracetam vs Oxiracetam to improve memory and cognitive impairment in patients with traumatic brain injury. Your condition may meet the inclusion criteria for this study, therefore, we would like to invite you to participate in this study. This informed consent form will introduce you to the purpose, steps, benefits, risks, inconvenience or discomfort of the study, etc. Please read carefully and make a careful decision on whether to participate in the study. When the researcher explains and discusses the informed consent form with you, you can ask questions at any time and let him/her explain to you what you do not understand. You can make a decision after discussing with your family, friends, and your doctor.

If you are currently participating in other clinical studies, please inform your research doctor or research staff.

The lead unit of this study is Tianjin Medical University General Hospital, and the project is led by Director Rongcai Jiang. The sponsor of this study is Nanjing Youke Pharmaceutical Co., Ltd.

**Why is this study being conducted?**

(I) Relevant Background

With the development of modern industry, power machinery, and high-speed transportation tools, the incidence of traumatic brain injury is increasing, and cognitive impairment is one of the most common and persistent sequelae of traumatic brain injury. Due to the complex mechanism of acute brain injury and the difficulty of treatment, the world is exploring its treatment to promote effective recovery from brain injury. Countries in Europe, mainly Italy, have carried out research and application of nootropic drugs. This is a new type of central nervous system drug that can promote learning and memory ability. The most notable is the pyrrolidone class, its representative drug Oxiracetam, was first synthesized by the Italian company in 1974, first listed in Italy in 1987, and then listed in Portugal in 1991. In 2003, the China Food and Drug Administration approved Oxiracetam capsules for domestic listing, and in 2005 and 2010, Oxiracetam injection and Oxiracetam for injection were approved for domestic listing, respectively.

However, to date, countries around the world have different attitudes towards the use of brain injury recovery drugs. The United States and Canada do not advocate the use of any drugs to promote brain injury repair; some patients with brain trauma in Europe receive nootropic drug treatment. In our country, Oxiracetam has become a commonly used neuroprotective drug for patients with nerve injury, and some patients with brain trauma are also using it. It can be confirmed that the use of Oxiracetam rarely causes toxic side effects, and it can effectively promote brain injury recovery in animal experiments, but there is no standardized clinical research to confirm its obvious effectiveness. The drug in our study - Oxiracetam for injection, is a single levorotatory enantiomer of Oxiracetam. Preclinical studies have confirmed that the application of L-oxiracetam may reduce the clinical use dose of Oxiracetam, thereby further reducing the potential toxic side effects of Oxiracetam.

In view of the different uses of nootropic drugs in acute brain injury in Europe and the United States, and there is no actual evidence to prove that patients with brain trauma must use nootropic drugs, this study plans to use a placebo as a control for whether Oxiracetam and L-oxiracetam are effective and safe. And it has obtained the approval of the National Drug Administration and the Ethics Committee.

(II) Purpose of the Study

The purpose of this study is to evaluate the effectiveness and safety of L-oxiracetam in improving memory and cognitive impairment in patients with acute traumatic brain injury.

**Who can be invited to participate in this study?**

If you meet the following criteria: ① Age is 18-75 years old (including boundary values); ② Traumatic brain injury meets the following conditions: This diagnosis has a clear head injury, closed traumatic brain injury, or traumatic brain injury accompanied by cerebrospinal fluid otorrhea and/or rhinorrhea and/or intracranial pneumocephalus; MRI or CT confirmed that there is intracranial hemorrhage above the cerebellar tentorium (including cerebral contusion, subarachnoid hemorrhage, epidural hematoma, subdural hemorrhage, intracerebral hematoma, etc.), with or without transient coma; The injury of traumatic brain injury is light and medium (GCS score 9-15 points); The condition is stable within 72 hours after traumatic brain injury, only conservative treatment is needed, and craniotomy is not required (there can be non-general anesthesia or non-basic anesthesia intracranial pressure monitoring of brain parenchyma); ③ Simple mental state examination, that is, MMSE score is lower than normal; then you will be invited to participate in this study.

If you meet any of the following conditions, you will not be able to participate in this study. ① Known or suspected allergy to the test drug and its components. ② After the injury, drugs that improve cognitive function listed in the plan have been used. ③ There is a history of severe traumatic brain injury, cerebrovascular accident, or structural cranial brain lesion. ④ There are diseases such as speech/hearing impairment that cannot cooperate to complete cognitive function assessment. ⑤ Secondary brain injury occurred after this traumatic brain injury. ⑥ Need to undergo craniotomy or ventricular drainage. ⑦ Combined with other serious organ injuries or serious complications that may affect the life of the subject. ⑧ Patients with active epilepsy within 1 year. ⑨ Combined with severe liver and kidney disease. ⑩ Combined with severe heart disease, lung disease, blood and hematopoietic system disease, gastrointestinal disease or other serious or progressive diseases. ⑪ Past or current malignant tumor (except for cured IB stage or lower cervical cancer, non-invasive basal cell or squamous cell skin cancer; breast cancer with complete remission (CR) >10 years, malignant melanoma with complete remission (CR) >10 years, other malignant tumors with complete remission (CR) >5 years).

⑫ Combined with neurological, mental diseases and unable to cooperate or unwilling to cooperate. ⑬ Pregnant, lactating women or those who have plans for childbirth in the near future. ⑭ The researcher believes that it is not suitable to participate in this clinical trial. ⑮ Participated in other clinical trials and used experimental drugs 3 months before the trial.

**How many people will participate in this study?**

This study will be conducted concurrently at multiple clinical research centers led by Tianjin Medical University General Hospital, and it is expected that 590 patients will voluntarily participate.

**How is this study conducted?**

This study will last for 14 days of medication, and you will need to follow up for 3 months after the end of the medication. If you voluntarily participate in this study, we hope you will cooperate with the following matters:

Your supervising doctor will inform you about the relevant situation of the study and answer all related questions. After signing the informed consent form, you will undergo a checkup including vital signs, and the doctor will also ask about your medical history and conduct a cognitive function assessment. You will also undergo routine blood tests (WBC, RBC, HGB, PLT), routine urine tests (PRO, GLU, LEU, ERY), liver function tests (AST, ALT, TBIL, γ-GT, ALP), kidney function tests (Scr, GFR), coagulation function tests (PT, APTT, TT, FIB), creatine kinase (CK), triglyceride (TG) and electrocardiogram tests, and women of childbearing age need to undergo a blood pregnancy test. All of these tests are free of charge. After screening, if you meet the inclusion criteria and do not meet the exclusion criteria, you will be randomly assigned (like flipping a coin) to the L-oxiracetam injection group, Oxiracetam injection group, or placebo group. You have a 1/5 chance of entering the placebo group. The specific medication method is shown in the table below.

In the routine treatment of traumatic brain injury to control intracranial hypertension and prevent complications:

| Experimental Group | L-oxiracetam for injection, 4 vials each time, once a day, intravenous drip.  Oxiracetam simulator for injection, 6 vials each time, once a day, intravenous drip. |
| --- | --- |
| Positive Control Group | L-oxiracetam simulator for injection, 4 vials each time, once a day, intravenous drip.  Oxiracetam for injection, 6 vials each time, once a day, intravenous drip. |
| Placebo Group | L-oxiracetam simulator for injection, 4 vials each time, once a day, intravenous drip.  Oxiracetam simulator for injection, 6 vials each time, once a day, intravenous drip. |

- During the medication process, if you have any discomfort or other problems, please inform your supervising doctor in time.

- After the end of the medication, you still need to cooperate with the doctor to complete routine blood tests (WBC, RBC, HGB, PLT), routine urine tests (PRO, GLU, LEU, ERY), liver function tests (AST, ALT, TBIL, γ-GT, ALP), kidney function tests (Scr, GFR), coagulation function tests (PT, APTT, TT, FIB), creatine kinase (CK), triglyceride (TG) and electrocardiogram tests, and conduct cognitive function and living ability assessments to understand the efficacy and safety of the drug. All these tests are free of charge.

- One month and two months after the end of the medication, the doctor will follow up with you (or your legal guardian) by phone to assess your daily living ability.

- Three months after the end of the medication, you need to come to the hospital for follow-up, accept necessary examinations including CT or MRI, vital signs, and cooperate with the doctor to complete routine blood tests (WBC, RBC, HGB, PLT), routine urine tests (PRO, GLU, LEU, ERY), liver function tests (AST, ALT, TBIL, γ-GT, ALP), kidney function tests (Scr, GFR), coagulation function tests (PT, APTT, TT, FIB), creatine kinase (CK), triglyceride (TG) and electrocardiogram tests, women of childbearing age need to undergo a blood pregnancy test. The doctor will also assess your cognitive function and living ability.

**What is the impact of participating in this study on the daily life of the subjects?**

When deciding whether to participate in this study, please carefully consider the possible impact of the above-listed examinations and follow-ups on your daily work, family life, etc. Consider the time and transportation issues for each return visit. If you have any questions about the examinations and procedures involved in the trial, you can consult us.

After the injury, cognitive rehabilitation treatment is not allowed. During the medication and follow-up period, the use of the following drugs that may affect the evaluation of efficacy is prohibited:

Cholinesterase inhibitors, such as Donepezil, Rivastigmine, Galantamine, Neostigmine, huperzine A, etc.;

γ-lactam brain function improvers, such as Aniracetam, Piracetam and other market-available Oxiracetam, etc.;

Other drugs: Nicergoline, Nimodipine, Ginkgo biloba preparations, cerebrolysin, Almitrine-raubasine, Ganglioside, Citicoline, Idebenone, Tyrosine, Safflower Extract and Aceglutamide Injection, Dihydroergosine mesylate, Brain protein hydrolysate, Mouse nerve growth factor, Pyrithioxin hydrochloride, Acetylglutamine, Acetamide pyrrolidone, 2-(2-Aminoethyl)isothiourea dihydrobromide, Hormone replacement therapy (growth hormone, estrogen, thyroid hormone), etc.

Before undergoing any treatment or taking any new medication, please consult your research doctor.

Considering your safety and to ensure the validity of the research results, you cannot participate in any other clinical research related to drugs and medical devices during the study period.

**What are the risks and adverse reactions of participating in this study?**

The clinical adverse reactions of Oxiracetam for injection include anxiety, skin itching, rash, nausea, stomach pain, etc., which can subside on their own after stopping the medication. A few patients have experienced mental excitement and sleep abnormalities. The side effects of L-oxiracetam are similar, and due to the lower dose, there may be fewer side effects. The incidence of the above side effects is very low, and they can recover on their own after stopping the medication.

The doctor will monitor drug side effects. During the study, if you experience any side effects or discomfort, you should immediately report to the research doctor.

The doctor may give you other drugs to control side effects. If you or your research doctor believe that you cannot tolerate these side effects, the research drug may be completely discontinued, and you may withdraw from this study.

Other risks associated with research such as:

**Reproductive risks**

For female subjects: This study does not recruit pregnant or lactating women. Since Oxiracetam and L-oxiracetam may affect reproduction, participants in this study who are sexually active must use contraception and should continue to use contraception until 3 months after the last administration of the research drug.

During the study, if you or your female partner become pregnant or think you may be pregnant, you should immediately tell the research doctor so that the study will not have reproductive accidents.

**Other risks**

There may also be some currently unpredictable risks, discomfort, drug interactions, or adverse reactions.

If personal private information is inadvertently leaked, it may have a negative impact on your work, study, and life.

**What are the possible benefits for the subjects participating in this study?**

By participating in this study, you will, to a certain extent, receive more attention from senior doctors, which is beneficial for the improvement of the condition; the results of laboratory tests conducted in the study will also help you and the doctor make judgments about your own health status. At the same time, we hope that the information obtained from your participation in this study can benefit patients with the same condition as you in the future.

**Are there other alternative treatment options if not participate in this study?**

You can choose not to participate in this study, which will not have any adverse effects on your routine treatment. Currently, for your health condition, similar nootropic drugs have been used in clinical treatment, such as Piracetam, Aniracetam, etc. The treatment plan of this study is not the only treatment option for your current disease, you can discuss with your doctor and then decide whether to participate in this study.

**Is it necessary to participate and complete this study?**

Your participation in this study is entirely voluntary. If you do not wish to, you can refuse to participate, which will not have any negative impact on your current or future health care. Even if you agree to participate, you can change your mind at any time, tell the researcher to withdraw from the study, you will not be discriminated against or retaliated against for withdrawing from the trial, and it will not affect your access to normal medical services. When you decide not to participate in this study anymore, we hope you will inform your research doctor in time, and the research doctor can provide advice and guidance on your health condition.

The sponsor or regulatory agency may also terminate this study during the study period. If this study is terminated prematurely, we will notify you in time, and your research doctor will provide advice for your next treatment plan based on your health condition.

For patients who drop out halfway, for safety reasons, we have a last follow-up plan, and you have the right to refuse. If after you withdraw, new information related to your health and rights is found, we may contact you again.

In principle, after you withdraw, the researcher will strictly keep your relevant information until it is finally destroyed, and will not continue to use or disclose this information during this period. But in the following rare cases, the researcher will continue to use or disclose your relevant information, even if you have withdrawn from the study or the study has ended. These situations include: removing your information will affect the scientific nature of the research results or the evaluation of data safety; providing some limited information for research, teaching or other activities (this information will not include your name, ID number, or other personal information that can identify you); when government regulatory agencies need to supervise the study, they will request to view all research information, which will also include your relevant information when you participated in the study.

**The costs of participating in this study**

The sponsor, Nanjing Youke Pharmaceutical Co., Ltd., will pay for the examination fees related to the study during your participation in this study, the registration fee during follow-up, provide research drugs for free, and provide a follow-up subsidy of a total of 600 yuan (including 100 yuan for each telephone follow-up after discharge, a total of two times; 400 yuan for the final visit to the hospital), which will be issued after the group is disbanded.

If the patient suffers damage such as adverse reactions or serious adverse reactions related to the study, the sponsor will bear the corresponding treatment costs and economic compensation.

If you need treatment and examination for other diseases at the same time, it will not be free.

**How is research-related injury handled?**

If your health condition is harmed during your participation in this study, please inform the researcher, and we will take necessary medical measures. According to the relevant laws and regulations in our country, when a research-related injury occurs, the sponsor of this study will bear the corresponding medical expenses and provide corresponding economic compensation.

**What do I need to do if I participate in the study?**

- Provide accurate past medical history and current health information.

- Inform the doctor of any health problems that occur during the study.

- Tell the doctor about any treatment you have undergone during the study and any new drugs, medications, vitamins, or herbs you have taken.

- Unless permitted by a doctor, you should not take any medication or treatment, including prescription drugs and drugs purchased over the counter at pharmacies (including vitamins and herbs).

- Take the research medication as directed by the doctor and visit as required.

- Do not participate in other medical research.

- Take contraceptive measures acceptable to the doctor.

- Follow the guidance of researchers and research doctors.

- You can ask at any time if there is anything unclear.

**Will the personal information of the subjects be kept confidential?**

If you decide to participate in this study, your participation in the study and your personal information in the study will be kept confidential. Your blood/urine samples will be identified by the research number rather than your name. Information that can identify you will not be disclosed to members outside the research team unless your permission is obtained. All research members and research sponsors are required to keep your identity confidential. Your files will be kept in a locked file cabinet for researchers to view. To ensure that the research is conducted in accordance with regulations, when necessary, members of government management departments or ethics committees can view your personal information in the research unit as stipulated. When the results of this study are published, your personal information will not be disclosed.

**What about new information related to the study?**

During the trial, we may learn new information about the treatment, and we will notify you in time to let you decide whether to continue to participate in the study or withdraw.

**Will the research drug treatment continue after the study ends?**

After the end of the study, Nanjing Youke Pharmaceutical Co., Ltd. (the sponsor) will no longer continue to provide you with research drugs. Your doctor will discuss your future treatment plan with you.

**Who should I contact if I have questions or difficulties?**

If you have any questions related to this study, please contact the doctor _______, contact number: _______ .

If you have questions related to the rights of the subjects themselves, you can contact _______ Medical Ethics Committee of the hospital, contact number: _______.

# Informed Consent Form · Patient Signature Page

Subject's Informed Consent Statement

I have been informed about the research background, purpose, steps, risks, and benefits of the L-oxiracetam injection project. I have had enough time and opportunity to ask questions, and I am satisfied with the answers to the questions. I have also been informed that when I have questions or want to get more information, I should contact whom. I have read this informed consent form and agree to participate in this study. I know that I can withdraw from this study at any time during the study without any reason. I have been informed that I will receive a copy of this informed consent form, which contains my signature and the researcher's signature.

Subject's Signature: Date: Contact Number:

Guardian's Signature: Date:

Relationship with the Subject: Contact Number:

Note: When the subject or his/her guardian cannot read or write, at least one impartial witness must be present. The impartial witness must witness the entire process of the informed consent discussion and sign.

I confirm that the information in the informed consent form has been correctly explained and the subject and/or the subject's legal representative understand these information. The subject voluntarily agrees to participate in this study.

Impartial Witness Signature [if applicable] Date: Contact Number:

Researcher's Notification Statement

I have informed the subject (and/or his/her guardian) about the research background, purpose, steps, risks, and benefits of the L-oxiracetam injection project, given him/her enough time to read the informed consent form, discuss with others, and answered his/her questions about the research; I have informed the subject of the contact information when encountering problems; I have informed the subject (and/or his/her guardian) that he/she can withdraw from this study at any time during the study without any reason.

Researcher's Signature: Date: Contact Number:
