# Supplementary material for: Effect of l‐oxiracetam and oxiracetam on memory and cognitive impairment in mild‐to‐moderate traumatic brain injury patients: Study protocol for a randomized controlled trial
Source: Aging Med (Milton). 2024 Jun 14;7(3):341–9. doi: 10.1002/agm2.12335 (PMC11222749; doi:10.1002/agm2.12335)
Supplement: Supplementary file 3 — Appendix S3. [file AGM2-7-341-s001.docx]

**Appendix 3. Prohibited treatment and medication**

**Treatment**

Cognitive rehabilitation therapy is not allowed after injury.

**Medication**

The following medications, which may affect the evaluation of efficacy, are not allowed during the treatment period and follow-up period:

1. cholinesterase inhibitors, such as donepezil, rivastigmine, galantamine, memantine, huperzine A, etc.
2. γ-lactams, such as aniracetam, piracetam, and other commercially available oxiracetams, etc.
3. hormone replacement therapy, such as growth hormone, estrogen, thyroid hormone, etc.
4. Other drugs, such as nicergoline, nimodipine, extracts of ginkgo biloba, cerebrolysin, almitrine/raubasine, gangliosides, citicoline, idebenone, aminobutyric acid, Guhong injection, dihydroergotoxine methanesulfonate, cerebroprotein hydrolysate, mouse nerve growth factor, pyrithioxine hydrochloride, aceglutamide, piracetam, aminethronii hydrobromidum, etc.
