# Supplementary material for: Effect of l‐oxiracetam and oxiracetam on memory and cognitive impairment in mild‐to‐moderate traumatic brain injury patients: Study protocol for a randomized controlled trial
Source: Aging Med (Milton). 2024 Jun 14;7(3):341–9. doi: 10.1002/agm2.12335 (PMC11222749; doi:10.1002/agm2.12335)
Supplement: Supplementary file 4 — Appendix S4. [file AGM2-7-341-s004.docx]

**Appendix 4. Scoring Criteria Attachments**

**Attachment 1.** **Mini-Mental State Examination (MMSE)**

| Item | | | Score | | | | | |
| --- | --- | --- | --- | --- | --- | --- | --- | --- |
| I Orientation  (10 point) | What year is it now? | |  |  |  |  | 1 | 0 |
|  | What season is it now? | |  |  |  |  | 1 | 0 |
|  | What month is it now? | |  |  |  |  | 1 | 0 |
|  | What date is it today? | |  |  |  |  | 1 | 0 |
|  | What day of the week is it today? | |  |  |  |  | 1 | 0 |
|  | Which county (district) do you live in? | |  |  |  |  | 1 | 0 |
|  | Which province do you live in? | |  |  |  |  | 1 | 0 |
|  | Which town (street) do you live in? | |  |  |  |  | 1 | 0 |
|  | Which hospital are we in now? | |  |  |  |  | 1 | 0 |
|  | What floor are we on now? | |  |  |  |  | 1 | 0 |
| II Memory  (3 point) | I will tell you three things, please repeat them after me and remember them, I will ask you again later (1 point each, total 3 points) | |  |  | 3 | 2 | 1 | 0 |
| III Attention and Calculation  (5 point) | I would like you to count backward from 100 by sevens.Subtract 5 times continuously (93, 86, 79, 72, 65. 1 point each, total 5 points. If wrong, but the next answer is correct, only count one error) | | 5 | 4 | 3 | 2 | 1 | 0 |
| IV Recall  (3 point) | Now, can you tell me the three things I just told you to remember? | |  |  | 3 | 2 | 1 | 0 |
| V Language Ability  (9 point) | Naming Ability | Show a watch, ask the patients to name it. |  |  |  |  | 1 | 0 |
|  |  | Show a pen, ask the patient to name it. |  |  |  |  | 1 | 0 |
|  | Repetition Ability | I will say a sentence now, please repeat it clearly after me (sì shí sì zhī shí shī zi) |  |  |  |  | 1 | 0 |
|  | Reading Ability | (Close your eyes) Please read this sentence and do as it says. |  |  |  |  | 1 | 0 |
|  | Three-step Command | I will give you a piece of paper, please do as I say, start now: “Take the paper in your right hand, fold it in half with both hands, and place it on your left leg.” (1 point for each action, total 3 points) |  |  | 3 | 2 | 1 | 0 |
|  | Writing Ability | Make up and write a complete sentence about anything. |  |  |  |  | 1 | 0 |
|  | Structural Ability | Please copy this picture. |  |  |  |  | 1 | 0 |
| Total Score |  | | | | | | | |

**Operating Instructions**

I. Orientation (Maximum: 10 points)

First, ask about the date, then ask other parts specifically, such as "Can you tell me what season it is now?", each correct answer gets one point. Please ask in order, "Can you tell me which province you live in?" (county, street, where, which floor), each correct answer gets one point.

II. Memory (Maximum: 3 points)

Tell the testee that you will ask a few questions to check his/her memory, then clearly and slowly say the names of 3 unrelated things (such as: ball, national flag, tree, about 1 second for each). After saying all 3 names, ask the testee to repeat them. The testee's score depends on their first repetition of the answer. (1 point for each correct answer, up to 3 points). If they can't remember all, you can repeat, but the number of repetitions cannot exceed 5 times. If they still can't remember all 3 names after 5 times, then the check for recall ability is meaningless. (Please skip part IV "Recall Ability" check).

III. Attention and Calculation (Maximum: 5 points)

Ask the patient to start from 100 and subtract 7, then subtract 7 again, subtract 5 times in total (i.e., 93, 86, 79, 72, 65). Each correct answer gets 1 point, if the previous one was wrong, but the next answer is correct, also get 1 point.

IV. Recall Ability (Maximum: 3 points)

If the testee completely remembered the 3 names last time, now let them repeat them again. Each correct repetition gets 1 point, up to 3 points.

V. Language Ability (Maximum: 9 points)

1. Naming Ability (0-2 points): Show the watch card to the testee and ask them what this is? Then show the pen and ask them the same question.

2. Repetition Ability (0-1 point): Ask the testee to pay attention to what you say and repeat it once, note that only one repetition is allowed. This sentence is "sì shí sì zhī shí shī zi", only correct, clear pronunciation can score 1 point.

3. Three-step Command (0-3 points): Give the testee a blank piece of paper, ask them to do as you command, note not to repeat or demonstrate. Only the actions they do in the correct order are correct, each correct action scores 1 point.

4. Reading Ability (0-1 point): Show a "Close your eyes" card to the testee, ask the testee to read it and do as required. Only if they actually close their eyes can they score.

5. Writing Ability (0-1 point): Give the testee a piece of white paper, let them spontaneously write a complete sentence. The sentence must have a subject, a verb, and make sense. Note that you cannot give any hints. Grammar and punctuation errors can be ignored.

6. Structural Ability (0-1 point): On a piece of white paper, there are two intersecting pentagons, ask the testee to draw them accurately. Evaluation criteria: The pentagon needs to draw 5 clear angles and 5 sides. At the same time, the intersection of the two pentagons forms a diamond, the jitter of the lines and the rotation of the figure can be ignored.

Attachment 2. Montreal Cognitive Assessment (MoCA）

| Visual Space and Executive Function | | | | | | | | | | | | | | | | | | | | | | | Score |
| --- | --- | --- | --- | --- | --- | --- | --- | --- | --- | --- | --- | --- | --- | --- | --- | --- | --- | --- | --- | --- | --- | --- | --- |
|  | E A  5 End  2  B  1  Start  D 4  3  C | | | | | | Copy Cube | | | | Draw Clock(**10 past 11**  ）(3 point) | | | | | | | | | | | | /5 |
|  |  |  |  | [ ] |  |  |  |  |  | [ ] | Outline[ ] Pointer[ ] Number[ ] | | | | | | | | | | | |  |
| Naming | | | | | | | | | | | | | | | | | | | | | | |  |
| 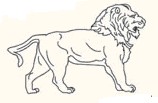 | | |  | 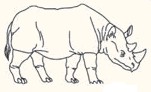 | | | | | |  |  | 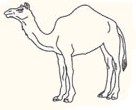 | | | | | | | | | | | /3 |
|  |  | [ ] |  |  |  |  |  |  | [ ] | |  |  | |  | | | |  |  | | [ ] | |  |
| Memory | | Read the following words, then have the patient repeat the process 2 times, recall after 5 minutes. | |  | | Face | | | Velvet | | Church | | | | Chrysanthemum | | | | | | | Red | Not counted |
|  |  |  |  | First time | |  | | |  | |  | | | |  | | | | | | |  |  |
|  |  |  |  | Second time | |  | | |  | |  | | | |  | | | | | | |  |  |
| Attention | | Read the following numbers, please have the patient repeat (1 per second). | | | | | | | | | | | Forward[ ] | | | | | | | **21854** | | | /2 |
|  |  |  |  |  |  |  |  |  |  |  |  |  | Backward[ ] | | | | | | | **742** | | |  |
| Read the following numbers, every time the number 1 appears, the patient knocks on the table once, if the number of errors is greater than or equal to 2, do not give. | | | | | | | | [ ]52139411806215194511141905112 | | | | | | | | | | | | | | | /1 |
| Serail 7 subtraction starting at 100. | | | | [ ]93 | [ ]86 | | | | | [ ]79 | | | [ ]72 | | | | [ ]65 | | | | | | /3 |
| 4~5 correct give 3, 2~3 correct give 2, 1 correct give 1, all wrong is 0. | | | | | | | | | | | | | | | | | | | | | | |  |
| Language | | Language Repeat: I only know that Zhang Liang came to help today. [ ] When the dog is in the room, the cat always hides under the sofa [ ] | | | | | | | | | | | | | | | | | | | | | /2  /1 |
|  |  | Fluency:Say as many animal names as possible in 1 minute. [ ]  (N≥11 Name) | | | | | | | | | | | | | | | | | | | | |  |
| Abstract | | Similarity: Like Banana—Orange=Fruit [ ]Train—Bicycle [ ]Watch—Ruler | | | | | | | | | | | | | | | | | | | | | /2 |
| Delayed Recall | | Cannot prompt when recalling | | Face  [ ] | | Velvet  [ ] | | | Church  [ ] | | Chrysanthemum [ ] | | | | | Red  [ ] | | | | Only based on non-prompted memory | | | /5 |
|  |  | Class prompt: | |  | |  | | |  | |  | | | | |  | | | |  |  |  |  |
|  |  | Multiple-choice prompt: | |  | |  | | |  | |  | | | | |  | | | |  |  |  |  |
| Orientation | | Date[ ] Month[ ] Year[ ] Day of the week[ ] Place[ ] City[ ] | | | | | | | | | | | | | | | | | | | | | /6 |
| Total Score | |  | | | | | | | | | | | | | | | | | | | | | /30 |

**MoCA Use and Evaluation Guide**

**1:Alternating Line Test**

Instructions: "Sometimes we use '123...' or English 'ABC...' to indicate order. Please draw a line in order from number to English character and gradually increasing. Start here [point to number (1)], from 1 to A, then to 2, and keep going until it ends here [point to English character (E)]."

Evaluation: When the patient completely follows the order of "1-A-2-B-3-C-4-D-5-E" for the line and there are no crossed lines, give 1. When the patient makes any mistakes and does not immediately correct them, give 0.

**2:Visual Structural Skills (Cube)**

Instructions (examiner points to the cube): "Please draw this picture again in the blank space below as accurately as possible."

Evaluation: When it fully meets the following standards, give 1: The figure is

three-dimensional

All lines are present

with no extra lines

Relative sides are basically parallel, length is basically consistent (rectangle or prism is also correct)

If any of the above standards are violated, it is 0.

**3:Visual Structural Skills (Clock)**

Instructions: "Please draw a clock here, fill in all the numbers and indicate 10 past 11." Evaluation: When it meets the following three standards, give 1:

Outline (1 point): The surface must be a circle, allowing minor defects (such as, the circle is not closed).

Numbers (1 point): All numbers must be complete and no extra numbers; the order of numbers must be correct and within their quadrant; Roman numerals can be used; numbers can be placed outside the circle.

Pointer (1 point): There must be two pointers and they point to the correct time together; the hour hand must be significantly shorter than the minute hand; the center intersection of the pointers must be inside the clock and close to the center of the clock.

If any of the above items are violated, do not give points for that item.

**4:Naming**

Instructions: From left to right, ask the patient while pointing at the picture: "Can you tell me the name of this animal?"

Evaluation: Give 1 for each correct answer. The correct answers are: (1) Lion; (2) Rhino; (3) Camel or Dromedary.

**5:Memory**

Instructions: The examiner reads 5 words at a rate of 1 word per second and tells the patient: "This is a memory test. In the following time, I will read you a few words, you need to listen carefully, you must remember. When I finish reading, tell me the words you remember. When answering, say whatever you think of, you don't have to follow the order I read." Mark the words the patient answered correctly in the first trial column. When the patient has answered all the words, or can no longer recall, read these 5 words again, and tell the patient: "I will read these words again, try to remember and tell me the words you remember, including the words you have already said in the first time." Mark the words the patient answered correctly in the second trial column.

After the second trial ends, tell the patient that he will be asked to recall these words later: "At the end of the examination, I will ask you to recall these words again."

Evaluation: These two recalls are not scored.

**6: Attention**

Number Forward Span: Instructions: "Now I'm going to say some numbers, you listen carefully, when I finish, you repeat them in the same way." Read these 5 numbers at a rate of 1 number per second.

Number Backward Span: Instructions: "Now I'm going to say some more numbers, you listen carefully, but when I finish, you must repeat them in reverse order." Read these 5 numbers at a rate of 1 number per second.

Evaluation: Accurate repetition, give 1 for each number series (note: the correct answer for backward is 2-4-7).

Alertness: Instructions: The examiner reads the number string at a rate of 1 per second and tells the patient: "Now I'm going to read a series of numbers, please listen carefully. Every time I read 1, you clap your hands. Don't clap your hands when I read other numbers."

Evaluation: If it is completely correct or only one mistake is made, give 1, otherwise do not give (error is when the patient does not clap when reading 1, or claps when reading other numbers).

Continuous Minus 7: Instructions: "Now please do a calculation problem, subtract a 7 from 100, then subtract another 7 from the result, keep subtracting until I ask you to stop." If necessary, you can explain to the patient again.

Evaluation: This item totals 3. All wrong scores 0, one correct scores 1, two to three correct scores 2, four to five correct scores 3. From 100 start to calculate the correct subtraction, each subtraction is evaluated separately, that is to say, if the patient subtracts wrong once, and all the subsequent subtractions of 7 are correct, then the subsequent correct subtractions should be given. For example, if the patient's answer is 93-85-78-71-64, 85 is wrong, but all other results are correct, so give 3.

**7: Sentence Repetition**

Instructions: "Now I'm going to say a sentence to you, after I finish, please repeat what I said as exactly as possible [pause for a while]: I only know that Zhang Liang came to help today." After the patient answers, "Now I'm going to say another sentence, after I finish, please also repeat it as exactly as possible [pause for a while]: When the dog is in the room, the cat always hides under the sofa."

Evaluation: Correct repetition, give 1 for each sentence. The repetition must be accurate. Pay attention to the omission (such as, omitting "only", "always") and replacement/addition (such as "I only know that Zhang Liang..." said as "I only know Zhang Liang today..."; or "room" said as "house", etc.) in the repetition.

**8: Word Fluency**

Instructions: "Please say as many animal names as you know as quickly and as much as possible. The time is 1 minute, please think about it, are you ready? Start." Stop after 1 minute.

Evaluation: If the patient says ≥11 animal names within 1 minute, give 1. At the same time, record the patient's answer content on the back of the check table or on both sides. Dragon, Phoenix, Kirin and other deified animals are also correct.

**9: Abstract:**

Let the patient explain in what way each pair of words are similar, or what they have in common. Start with the example words. Instructions: "Can you tell me in what way oranges and bananas are similar?" If the patient's answer is a specific feature (such as,

Both have skin, or both can be eaten, etc.), then you can only prompt once: "Please change another way, in what way are they similar?" If the patient still does not give an accurate answer (fruit), then say: "What you said is not wrong, you can also say they are both fruits." But do not give any other explanation or explanation.

After the exercise is over, say: "Can you tell me in what way trains and bicycles are similar?" After the patient answers, proceed to the next group of words: "Can you tell me in what way watches and rulers are similar?" Do not give any other explanation or inspiration.

Evaluation: Only evaluate the answers to the last two groups of words. Correct answer, give 1 for each group of words. Only the following answers are considered correct:

Train and bicycle: transportation tool; travel tool. Watch and ruler: measuring instrument; used for measurement.

The following answers cannot be given:

Train and bicycle: both have wheels. Watch and ruler: both have numbers.

**10: Delayed Recall**

Instructions: "Just now I read you a few words to remember, please try to recall again, tell me what these words are?" Mark the words that are correctly recalled without prompting in the blank column below.

Evaluation: Correctly recall the words without prompting, give 1 for each word.

Optional Items:

After the delayed free recall, for words that cannot be recalled, encourage the patient to recall as much as possible through semantic Classification cues.

For those who recall correctly through Classification prompts or multiple-choice prompts, mark a check (√) in the corresponding blank column. If they still can't recall after the Classification prompt, proceed with the multiple-choice prompt. For example: "Which of the following words is one that you remembered: nose, face, palm?"

The Classification prompts and/or multiple-choice prompts for each word are as follows:

Scoring: Cue recall is not scored. Cue recall is only used for clinical purposes, providing further information for the examiner to analyze the type of memory impairment in the patient. For memory deficits caused by retrieval impairment, cues can improve recall performance; if it is encoding impairment, then cues do not help improve recall performance.

Red, Blue, Green

Red: A type of color

Rose, Chrysanthemum, Peony

Chrysanthemum: A type of flower

Church, School, Hospital

Church: A building

Cotton cloth, Dacron, Velvet

Velvet: A type of textile

Nose, Face, Palm

Face: Part of the body

Multiple-choice Prompt

Classification Prompt

**11:Orientation**

Instructions: "Tell me what today's date is." If the patient's answer is incomplete, you can prompt the patient: "Tell me what it is now [which year, which month, the exact date today, which day of the week]." Then ask again: "Tell me what this place is, and which city it is in?"

Evaluation: Give 1 for each correct answer. The patient must answer the exact date and location (name of the hospital, clinic, office). If the date is one day more or one day less, it is considered wrong, and no points are given.

Attachment 3. Loewenstein Occupational Therapy Cognitive Assessment (LOTCA)

**Loewenstein Occupational Therapy Cognitive Assessment (LOTCA)**

| Assessment Items | Method Summary |
| --- | --- |
| A.Orientation | |
| 1. Orientation to place | Ask the patient about their current location, city, home address, and where they stayed before admission. |
| 1. Orientation to time | Ask the patient about the day of the week, month, year, season, estimate the current time without looking at the clock, and how long they have been in the hospital. |
| B.Visual Perception | |
| 1. Object Identification | Let the patient identify 8 kinds of daily necessities pictures through naming, understanding, approximate pairing, and identical pairing: chair, teapot, watch, key, shoe, bicycle, scissors, glasses. |
| 1. Shape Identification | Let the patient recognize 8 different shapes of geometric figures through naming, understanding, approximate pairing, and identical pairing: square, triangle, circle, rectangle, diamond, semicircle, trapezoid, and hexagon. |
| 1. Overlapping Figures | Let the patient recognize the overlapping figure of banana, apple, pear; pliers, saw, hoe. |
| 1. Object Consistency | Let the patient identify 4 pictures of objects taken from special angles: car, hammer, telephone, and fork. Give the front windshield of the car, the back of the telephone, the side of the fork, the side of the hammer. |
| C. Spatial Perception | |
| 7. Body Orientation | Let the patient stretch out their right hand and left foot in turn; touch the opposite ear and thigh with their hand. |
| 8. Spatial Relationship with Surrounding Objects | Let the patient point out four different objects in four different directions in the room: front, back, left, and right. |
| 9. Spatial Relationship in Pictures | Show the patient a picture, then say the names of the objects in front, behind, left, and right of the character in the picture. |
| D. Praxis | |
| 10. Motor Imitation | Let the patient imitate the examiner's actions. |
| 11. Utilization of Objects | Let the patient demonstrate how to use 4 groups of objects: comb, scissors and paper, envelope and paper, pencil and eraser. |
| 12. Symbolic Actions | Let the patient demonstrate how to brush teeth, open the door with a key, cut bread with a dinner knife, make a phone call. |
| E. Visuomotor Organization | |
| 13. Copying Geometric Forms | Let the patient copy a circle, triangle, diamond, cube, and a composite figure. |
| 14. Reproducing a Two-dimensional Model | Let the patient draw geometric figures according to the given pattern, including a circle, a rectangle (square), two triangles, and some related shapes. |
| 15. Constructing a Pegboard Design | Let the patient insert pegs on the plastic pegboard to create the corresponding figure according to the given pattern. |
| 16. Constructing a Colored Block Design | Let the patient use colored blocks to piece together the corresponding three-dimensional figure according to the given pattern. |
| 17. Constructing a Plain Block Design | Ask the patient to piece together a three-dimensional figure according to the given pattern using colorless blocks, and state how many blocks are needed. |
| 18. Reproducing a Puzzle | Ask the patient to piece together a colored butterfly using 9 pieces of pattern fragments according to the given pattern. |
| 19. Drawing a Clock | Ask the patient to draw a clock on a piece of paper with a circle, mark the numbers, and indicate the position of the hour and minute hands at 10:15. |
| F. Thinking Operation | |
| 20. Item Classification | Ask the patient to categorize and name 14 items provided (sailboat, helicopter, airplane, bicycle, ship, train, car, hammer, scissors, needle, screwdriver, sewing machine, hoe, rake) according to different principles. |
| 21. Riska Object Classification (Unstructured) | Ask the patient to categorize 18 plastic pieces of three different colors (dark brown, light brown, cream) and three different shapes (arrow, ellipse, 1/4 sector) according to a certain intention (such as color or shape). |
| 22. Riska Object Classification (Structured) | Similar to 21, the difference is that the patient categorizes 18 plastic pieces according to the categorization method demonstrated by the examiner. |
| 23. Picture Sequence A | Give the patient 5 pictures that are out of order but related in content, ask the patient to arrange them in a logical order, and describe the story plot. |
| 24. Picture Sequence B | Give the patient another 6 pictures that are out of order but related in content, ask the patient to arrange them in a logical order, and describe the story plot. |
| 25. Geometric Sequence | Show the patient a set of geometric figures that change according to a certain rule, ask the patient to continue arranging according to the arrangement rule of the figures. |
| 26. Logical Questions | Ask the patient to look at four logical questions (one at a time), and then answer. For example: Zhang Ming was born in 1930, in which year should he be 35 years old? Xiao Li has 5 apples, Xiao Shan has 3 less than Xiao Li, how many apples do they have in total? |
| G. Attention and Concentration | |
| Evaluate the patient's attention and concentration during the entire evaluation process. | |

**LOTCA Operation and Scoring Standards**

I. Instructions for Use

1. Before using LOTCA, please carefully read the descriptions and correct operation methods for each test.

2. The scores for most test items range from 1 point (lowest) to 4 points (highest), except for the -F test items:

① The three item classification test items are scored from 1 point (lowest) to 5 points (highest); ② The two orientation test items are scored from 1 point (lowest) to 8 points (highest).

3. In each test, the test subject and the examiner should sit side by side, but the following test items should adopt a face-to-face seating method: ① Spatial perception items and ② Motor application items.

4. After each test item is finished, the examiner should ask the test subject: "Is this item completed?" and then score the test subject. The blank space behind each test item on the scoring form is used to fill in notes.

5. Patients with brain injuries are prone to fatigue. Some patients will express it when they are tired, but some are not aware of their own fatigue. Therefore, if the examiner finds that the test subject's actions are slowing down or restless, the test should be paused and continued after some time. At the end of the evaluation, the examiner should record the time required for the evaluation and whether the evaluation was completed in stages.

6. The examiner should objectively evaluate the level of attention and concentration of the test subject based on the observation of the test subject during the entire evaluation process.

II. Scoring Standards

A. Orientation

If the test subject's understanding ability is problematic (such as sensory aphasia), this item cannot be evaluated. If the test subject's comprehension is good but only has difficulty expressing, they can choose "yes" or "no" from the multiple choices provided by the examiner.

1. Orientation to plcae(OP)

Method: The examiner asks the test subject the following questions.

(1) Where are you now?

(2) What city are we in now?

(3) Where do you live? What is your exact address?

(4) Where were you before you came here?

For patients with language or memory impairments, multiple choices can be used. The examiner provides 3 options for the test subject to choose from, including one correct answer.

Scoring: The test subject gets 2 points for each correct answer; if they can only answer correctly after being given multiple choices, they get 1 point.

Minimum score: 1 point (all answers are wrong, or only one question is answered correctly after giving multiple choices):

Maximum score: 8 points (no need to give any options, all questions are answered correctly).

2. Orientation to time(OT)

Method: The examiner asks the test subject the following questions.

(1) What day of the week is it today? What month is it now? What year is it?

(2) What season is it now?

(3) What time is it now?

(4) How long have you been in the hospital? (If the test subject has not been hospitalized, the examiner can ask: "How long have you been sick?" or "How long have you been feeling unwell?")

For patients with language or memory impairments, multiple choices can be used. The examiner will provide 3 options for the test subject to choose from, including one correct answer.

Scoring: The test subject gets 2 points for each correct answer; if they can only answer correctly after being given multiple choices, they get 1 point.

Minimum score: 1 point (all answers are wrong, or only one question is answered correctly after giving multiple choices);

Maximum score: 8 points (no need to give any options, all questions are answered correctly).

B.Visual Perception

3. Object Identification(OI)

The object recognition cards (blue) in the test box and pages 1-4 of the test album are needed. Method:

● Naming: The examiner shows the test subject 8 cards of daily necessities: chair, teapot, watch, key, shoe, bicycle, scissors, glasses, and asks the test subject to say the name of each item. Note: Arrange the cards in the order provided above for questioning, do not use numbers on the back of the cards to arrange the order.

● Understanding: If the test subject has difficulty expressing and cannot say the name of the item, the examiner can open pages 1-2 of the test album, say the name of an item, and let the test subject point it out in the album. The examiner will ask: Which one is the chair; Which one is the watch; etc. Ask each of the 8 items one by one.

● Approximate Pairing: If the test subject has problems in understanding, the examiner takes out pictures similar to pages 1-2 of the test album, shows the test subject 8 cards one by one, and asks one by one: "Which one is this in the album?" The test subject is required to point out the item similar to a card in the album.

● Identical Pairing: If the test subject cannot distinguish similar objects, the examiner opens pages 3-4 of the album (this set of items is exactly the same as the items on the card). The examiner asks one by one: "Which one is this card in the album?" The test subject is required to pair the item on the card with the album.

Scoring:

1 point: Through the method of identical pairing, the test subject can only identify a few items (less than 4).

2 points: Through the method of identical pairing, the test subject can identify 5-8 items.

3 points: Through naming, understanding, and approximate pairing, the test subject can identify at least 4 items (4-7).

4 points: Through naming, understanding, and approximate pairing, the test subject can identify all items.

4. Shape Identification (SI)

The shape recognition cards (yellow) in the test box and pages 5-8 of the test album are needed. Method:

● Naming: The examiner shows the test subject 8 shapes on the cards one by one: square, triangle, etc. The test subject is asked to say the name of each shape.

● Understanding: If the test subject has difficulty expressing and cannot say the name of the shape, the examiner can open pages 5-6 of the test album and let the test subject point out the same shape on the album as the card. For example, "Please point out which shape is a circle", etc. The test subject is required to point out the corresponding shape.

● Approximate Pairing: If the test subject has problems in understanding and cannot recognize the shape, the examiner opens page 7 of the test album with similar shapes, shows the test subject 8 shape cards one by one, and asks the test subject to point out the shape on the album that is similar to the card.

● Identical Pairing: If the test subject cannot distinguish similar objects, then the examiner opens page 8 of the album. The shapes on page 8 are exactly the same as the shapes on the card. The examiner asks: "Which shape is this on the album?" The test subject is required to point out the matching item.

Note: The examiner shows the picture to the test subject in the exact same position as the shape on the test album. Scoring:

1 point: Through the method of identical pairing, the test subject can only identify a few items (less than 4).

2 points: Through the method of identical pairing, the test subject can identify 5-8 items.

3 points: Through naming, understanding, and approximate pairing, the test subject can identify at least 4 items (4-7).

4 points: Through naming, understanding, and approximate pairing, the test subject can identify all items.

Note: Arrange the cards and ask questions in the order provided above, do not use numbers on the back of the cards to arrange the order.

5. Overlapping Figures (OF)

The overlapping shape cards (green) in the test box and pages 9-10 of the test album are needed.

Method: The examiner shows the test subject two overlapping shape recognition cards, each recognition card has three objects overlapping: banana, pear, apple; pliers, hoe, saw.

The examiner asks the test subject: "What is drawn on the card?" If the test subject has difficulty recognizing geometric shapes, the examiner shows the test subject six individual item pictures on the test album, and then guides the test subject to answer: "Please point out in the album what you see on the card." The operation for the second card is the same.

Scoring:

1 point: Without the help of the album, the test subject cannot identify any items, or with the help of the album, the test subject can identify fewer than 3 items.

2 points: With the help of the album, the test subject can identify 3 items.

3 points: Without the help of the album, the test subject can identify at least 4 items, or with the help of the album, the test subject can identify all items.

4 points: Without the help of the album, the test subject can identify all items on the card.

6. Object Consistency (OC)

The test album's pages 11-19 are needed.

Method: The examiner shows the test subject four photos (page 11), the objects in the photos (car, hammer, telephone, and fork) are all taken from angles that are slightly different from what is normally seen, the examiner will ask the test subject for each photo: "What do you see in this photo?"

Only when the test subject has a language barrier (such as aphasia), the examiner can use multiple-choice pictures (pages 12-19). For example, let the test subject first look at the large picture on page 12, and then ask the test subject: "Please point out in these small pictures (page 13), the object you see in the large picture."

Each question only has one correct answer.

Scoring:

1 point: The test subject cannot identify any one object, or can only identify one of them.

2 points: The test subject can identify 2 objects.

3 points: The test subject can identify 3 objects.

4 points: The test subject can identify all 4 objects.

C. Spatial Perception

Method: The examiner and the test subject sit face to face.

7. Body Orientation

Method: The examiner can switch "left" and "right" according to the test subject's body problems. The examiner asks the test subject: (1) Stretch out your right hand.

(2) Stretch out your left foot.

(3) Put your right hand on your left ear.

(4) Put your left hand on your right thigh.

Scoring: Each correct response gets 1 point. The minimum score is 1 point, and the maximum score is 4 points.

8. Spatial Relationship with Surrounding Objects

Method: The examiner points out four different objects in four different directions (left, right, front, back) in the room to the test subject, and then asks the test subject:

(1) On which side of you? (For example: door)

(2) On which side of you? (For example: window)

(3) On which side of you? (For example: where I sit)

(4) On which side of you? (Any obvious object in the room)

Scoring: Each correct answer gets 1 point. The minimum score is 1 point, and the maximum score is 4 points.

9. Spatial Relationship in Pictures

The photo in the test box is needed.

Method: The examiner shows the test subject a photo, in which there is a man sitting in front of a table. The examiner asks the test subject:

(1) What is in front of this person?

(2) What is on the left side of this person?

(3) On which side of this person is the computer?

(4) What is behind this person?

Scoring: Each correct answer gets 1 point. The minimum score is 1 point, and the maximum score is 4 points.

D. Praxis

Motor application includes three groups of content: action imitation, use of objects, and symbolic actions.

10. Motor Imitation

Method: The examiner and the test subject sit face to face. The examiner tells the test subject: “Please imitate my actions, just like looking in a mirror.” If the test subject does not understand, the examiner can further explain with actions: “If I make a movement with my left hand, please make the same movement with your right hand.”

The examiner performs the following actions:

(1) Pinch the earlobe on the same side with the thumb and index finger of one hand.

(2) Continuous action: Put the palm on the back of the neck, and then put it on the shoulder on the opposite side.

(3) Put the back of one hand on the cheek on the opposite side (fingers straight).

(4) The thumb first touches the middle finger, then touches the ring finger, and repeats the above actions 3 times.

Scoring: Each correct response gets 1 point. The minimum score is 1 point, and the maximum score is 4 points.

Note: The mirror relationship in action imitation is mainly to test the use of actions, not the recognition of the left and right sides of the body. Therefore, whether the action is mirrored or on the opposite side, as long as the action is correct, full marks can be obtained.

11. Utilization of Objects

Method: The examiner shows the test subject one group of items at a time: a comb; a pair of scissors and a piece of paper; an envelope and a piece of paper; a pencil and an eraser. The examiner says to the test subject: "Please demonstrate how to use these items." When using the pencil and eraser, the examiner says to the test subject: "Please draw a straight line on the paper, and then erase it."

Scoring: Each correct response gets 1 point. The minimum score is 1 point, and the maximum score is 4 points.

12. Symbolic Actions (SA)

Method: The examiner asks the test subject:

(1) Please demonstrate to me how you brush your teeth. (The examiner asks the test subject to demonstrate the entire action, from applying toothpaste to the toothbrush, putting the toothbrush in the mouth, to the brushing action)

(2) Please demonstrate to me how you open the door with a key.

(3) Please demonstrate to me how you cut bread with a dinner knife.

(4) Please demonstrate to me how you make a phone call. (The examiner asks the test subject to demonstrate the entire action, from picking up the handset, dialing, to putting the handset to the ear)

Scoring: Each correct demonstration gets 1 point. The minimum score is 1 point, and the maximum score is 4 points.

E. Visuomotor Organization

In this part of the test, the test time needs to be recorded and filled in the score sheet.

13. Copying Geometric Forms (GF)

The geometric figure cards (orange) in the test box are needed.

Method: The examiner puts a piece of paper and a pencil in front of the test subject, and says to the test subject: "I will show you 5 figures, please draw these 5 figures on the paper." The examiner arranges the figures in the following order: circle, triangle, diamond, cube, and a composite figure.

Scoring: 1 point: Cannot draw any figure, or can only draw one of them.

2 points: Can draw 2 or 3 figures.

3 points: Can draw 4 figures.

4 points: Can draw 5 figures.

Note: When drawing a cube, the test subject must accurately draw the position of each edge of the figure to score. That is to say, the test subject should show a sense of three-dimensional space of the figure.

Please arrange the cards in the order provided above for questioning, do not use numbers on the back of the cards to arrange the order.

14. Reproducing a Two-dimensional Model

The 16th page of the test album is needed.

Method: The examiner shows the test subject a geometric pattern, including a circle, a rectangle (square), two triangles, and some related shapes. The examiner asks the test subject: "Draw this pattern next to this pattern." If the test subject cannot do this, the examiner can guide the test subject: "Draw directly on this pattern."

Scoring: 1 point: Cannot draw the pattern.

2 points: Can only draw directly on the pattern.

3 points: Can draw the pattern, but after repeated attempts and errors.

4 points: Can draw the pattern.

15. Constructing a Pegboard Design

The 17th page of the test album is needed.

Method: The examiner places the following tools in front of the test subject: a socket board, some plastic pegs, and the triangular pattern design on the 17th page of the test album. The examiner asks the test subject: "Use the pegs to complete the corresponding pattern on the socket board."

Scoring: 1 point: Cannot complete.

2 points: Can only complete vertical and horizontal lines, cannot complete diagonal lines and/or the pattern does not have corners.

3 points: Can complete the pattern, but the pattern is not correctly positioned on the socket board (see page 17).

4 points: Can correctly complete the pattern.

16. Constructing a Colored Block Design

The 18th page of the test album is needed.

Method: The examiner places the following tools in front of the test subject: 10 colored blocks and the pattern on the 18th page of the test album. The examiner asks the test subject: "Please piece together the model according to the pattern."

Scoring: 1 point: Cannot complete.

2 points: Can only establish a flat model on the table, without height or depth, or part of it is a flat model.

3 points: The established model only has height or only has depth.

4 points: Can correctly complete the puzzle.

17. Constructing a Plain Block Design

The 19th page of the test album is needed.

Method: The examiner places the following tools in front of the test subject: 10 five-color blocks and the pattern on the 19th page of the test album. The examiner asks the test subject:

1. How many blocks are needed to complete this model?

(2) Please start the puzzle.

Scoring: 1 point: Cannot complete the puzzle and cannot answer the number of blocks correctly.

2 points: Only the blocks that are seen are pieced together, ignoring the blocks that are not visible behind.

3 points: The number of blocks answered is wrong, but the model can be correctly pieced together: or the number of blocks answered is correct, but the model cannot be correctly pieced together.

4 points: Can correctly complete the puzzle.

18. Reproducing a Puzzle (RP)

The 20th page of the test album is needed.

Method: The examiner shows the test subject the colored butterfly pattern on the 20th page of the test album and the corresponding 9 pieces of pattern fragments, and asks the test subject to piece together the broken pictures on the pattern.

Scoring: 1 point: Cannot complete.

2 points: Can only piece together the vertical three fragments in the middle of the pattern.

3 points: After repeated attempts, the pattern can be correctly pieced together.

4 points: Can correctly piece together the pattern without needing to attempt.

19. Drawing a Clock

The 21st page of the test album is needed.

Method: The examiner gives the test subject a pencil and a piece of paper with a circle drawn on it (like the 21st page of the test album), and then says to the test subject: "Please write out the numbers inside the clock." Then draw the correct position of the hour hand and minute hand according to 10:15.

Scoring: 1 point: Cannot complete.

2 points: The clock face drawn roughly conforms to the intention, but the scale and the time marked are deviated.

3 points: The scale of the clock face drawn is correct, but the time marked is wrong: or the time marked is correct, but the position of the clock scale is incorrect.

4 points: Can correctly complete.

1. Cognitive Operations

20. Item Categorization

The item classification cards (red) in the test box are needed.

Method: The examiner randomly spreads 14 cards printed with the following items on the table: sailboat, helicopter, airplane, bicycle, ship, train, car, hammer, scissors, needle, screwdriver, sewing machine, hoe, rake.

Then the examiner asks the test subject: (1) Please group the cards by the type of items.

(2) Please name each group.

After the test subject completes the first grouping operation, the examiner will ask again:

(1) Could there be another way of classification?

(2) Please name each group according to the new classification method.

Scoring: 1 point: Cannot complete.

2 points: Can complete part of the item classification (can be coarse or fine).

3 points: Can complete the classification of items twice, but needs prompts and/or cannot complete all classifications.

4 points: Can complete the classification of items, can have or without prompts, but cannot summarize the classification standard in language.

5 points: Can complete the classification of items and can describe the classification standard in language. If the test subject cannot get the highest score due to language problems, the examiner should explain it next to the score on the scoring form.

21. Riska Object Classification (Unstructured)

Method: This test requires 18 plastic blocks in the test box with 3 different colors (dark brown, light brown, and cream) and 3 different shapes (arrow, ellipse, and 1/4 sector). All blocks are randomly placed in front of the test subject. The examiner says: "Group the objects you think are similar." After the test subject completes the grouping, the examiner asks the test subject: "Why are these grouped together?" (or: "What principle did you group by?") When the test subject describes its grouping standards, the examiner asks the test subject: "Now, group in another way."

Note: In order to keep consistent with the overall scoring standards of LOTCA, the scoring standards of the first edition of Riska Shape Classification are reduced.

Scoring:

1 point: Precise pairing (blocks of the same color and shape) and domain collection (arranging the blocks into a house or a flower pattern).

2 points: Classify according to an incomplete standard (for example, some blocks are missed and not grouped, or two standards are confused).

3 points: Arrange the blocks in three dimensions according to a standard (for example, arrange the oval blocks in a row, arrange the arrow blocks on the other side, etc.).

4 points: Randomly classify according to a standard, can switch from one standard to another (for example, first by color, then by shape).

5 points: Can classify according to two or more standards at the same time (for example, combine 2 or more shapes and colors in a group).

1. Riska Object Classification (Structured)

Method: The same plastic blocks as in test item 21 are needed. The examiner places a group in front of the test subject: a dark brown arrow, a cream-colored 1/4 sector, and a light brown ellipse. Then the examiner says to the test subject: "I have divided a group, now you start to divide a group similar to mine, try to divide as much as possible." If the test subject can divide all the blocks into groups, the examiner asks again: "What are the similarities between the group you divided and the group I divided?" If the test subject can say three different shapes and three different color standards, the test ends. If the test subject cannot say it, the examiner says again: "The group you divided is similar to the group I divided in some ways, but not in some ways, try to make them more similar." If some groups are not completed, the examiner can give the test subject a hint: "Use all the blocks."

Scoring:

1 point: Precise pairing (blocks of the same color and shape) and/or collection (arranging the blocks into a line or a flower pattern).

2 points: Classify according to an incomplete standard (for example, some blocks are missed and not grouped, or two standards are confused).

3 points: Classify according to a standard.

4 points: After the examiner's prompt, the second attempt can classify according to two standards at the same time.

5 points: The first attempt can classify according to two standards at the same time.

23 and 24 are picture sorting

The picture sorting story cards (A and B, both purple) in the test box are needed.

23. Picture Sequece A (PS1)

Method: The examiner spreads 5 cards in front of the test subject in the following order, which together form a small story.

5 2

4 1 3

The examiner asks the test subject:

(1) Please arrange the cards in the correct order.

(2) Please describe the plot of the story.

Scoring: 1 point: Cannot complete.

2 points: Only part of the cards are used, but it does not conform to the whole order.

3 points: Can describe the plot of the story, but the arrangement of the cards is incorrect; or the arrangement of the cards is correct, but cannot describe the plot of the story.

4 points: Correctly completed.

24. Picture Sequence B (PS2)

If the test subject scores 4 points in Picture Sorting A, or due to aphasia, can only arrange the cards in the correct order, then this picture sorting test is needed. The cards are spread out in the following order:

5 1 4

2 6 3

The requirements for the test subject and the scoring method are the same as in Picture Sorting A test. If the test subject cannot get full marks due to language problems, the examiner should note it on the scoring form. If the test subject scores less than 4 points in the Picture Sorting A test, then the Picture Sorting B test is not needed (this item has no points).

25. Geometric Sequence (GS)

The 26th and 27th pages of the test album are needed.

Method: The examiner shows the test subject the first set of geometric sorting shapes (the 26th page of the test album) and gives the test subject a pencil. The examiner says to the test subject: "In this column, these shapes are arranged in a specific order, please continue to draw according to this order." Repeat the test of the second set of sorting geometric shapes (the 27th page of the test album) in this way.

The correct answer to the first set of shape sequences is: circle, square. The correct answer to the second set of shape sequences is: four horizontal line segments, five vertical line segments. In the second set of shapes, if the test subject cannot understand the order of the shapes and cannot draw correctly (for example, the test subject continues to draw according to the beginning of the shape, or continues to draw part of the shape), then the examiner should guide the test subject: "Is there another possible way to continue this shape sequence?"

Scoring: 1 point: Cannot complete.

2 points: Only completes the continuation of the first set of shape sequences.

3 points: After several attempts, can complete the continuation of two sets of shape sequences,

4 points: Can correctly complete shape sorting.

Note: The test subject needs to draw at least two or more shapes that conform to the sorting to get 3 points.

26. Logical Questions (LQ)

Method: The examiner shows the test subject a page of paper with the following questions, and then reads them together. One question at a time. The test subject can choose to answer orally or in writing according to their own wishes. If the test subject has a language barrier, this test may be difficult.

Questions: (1) Zhang Ming was born in 1930, in which year should he be 35 years old?

(1) Li Da was born in 1950, how old is he this year?

(2) Xiao Li has 5 apples, Xiao Shan has 3 less than Xiao Li, how many apples do they have in total?

(3) Xiao Nan was born earlier than Xiao Zhen, but later than Xiao Sha. Who is the oldest? Who is in the middle? Who is the youngest?

Scoring: 1 point for each correct answer. The lowest score is 1 point, and the highest score is 4 points.

G. Attention and Concentration

Method: Score based on the performance of the test subject observed in the above test process.

Scoring:

1 point: The attention concentration period is very short, the test subject's attention does not exceed 5 minutes, and constant repetition is needed.

The test needs to be stopped (the entire test process cannot be completed at once).

2 points: The test subject can concentrate for a short time, the concentration exceeds 15 minutes; repeated prompts are needed. The entire test process needs to be completed in two times.

3 points: The test subject has slight difficulties in attention and concentration, but can still complete all test items after multiple re-concentrations.

4 points: There are no problems with attention and concentration.

Attachment 4. Glasgow Outcome Scale Extended (GOS-E)

**Glasgow Outcome Scale Extended (GOS-E)**

| Score | Characteristic |
| --- | --- |
| 8 | Upper Good Recovery, full recovery or minior symptoms that do not affect daily life |
| 7 | Lower Good Recovery, with minor physical or mental deficits that affects daily life |
| 6 | Upper Moderate Disability, some disabilitiy exists, but can partly resume work or previous activities |
| 5 | Lower Moderate Disability, independent but cannot resume work/school or previous social activities |
| 4 | Upper Severe Disability, needs partial assistance for daily activities |
| 3 | Lower Severe Disability, needs full assistance for daily activities |
| 2 | Vegetative State, absence of awareness of self and environment |
| 1 | Death |

Attachment 5. Glasgow Coma Scale (GCS)

**Glasgow Coma Scale (GCS)**

| Eye Opening Response | | Language Response | | Motor Response | |
| --- | --- | --- | --- | --- | --- |
| Item | Score | Item | Score | Item | Score |
| Open eyes spontaneously | 4 | Is oriented to person, place and time | 5 | Follows commands | 6 |
| Open eyes in response to speech | 3 | Converses, may be confused | 4 | Makes localized movement in response to painful stimulation | 5 |
| Open eyes in response to painful stimulations | 2 | Replies with inappropriate words | 3 | Makes non purposeful movements in response to painful stimulation (withdraws from pain) | 4 |
| Does not open eyes in response to any stimulation | 1 | Makes incomprehensible sounds | 2 | Flexes upper extremities / extends lower extremities in response to painful stimulation | 3 |
|  |  | Makes no response | 1 | Extends all extremities in response to painful stimulation | 2 |
|  |  |  |  | Makes no response to noxious stimulation | 1 |

Attachment 6. Activities of Daily Living (ADL) Ability Scale (Barthel Index)

Referencing the Activities of Daily Living (ADL) Ability Scale (Barthel Index) provided by the Disease Control Department of the Ministry of Health and the Neurology Branch of the Chinese Medical Association in the "China Stroke Prevention Guidelines" (trial)

Barthel Index scoring (total score is 100 points): ① Independent 100 points ② Mild dependence 75-95 points ③ Moderate dependence 50-70 points ④ Severe dependence 25-45 points ⑤ Total dependence 0-20 points

**Activities of Daily Living (ADL) Ability Scale**

| Daily Activity Item | Independent | Partially Independent or Requires Some Assistance | Requires Significant Assistance | Completely Dependent | Score |
| --- | --- | --- | --- | --- | --- |
| Eating | 10 | 5 | 0 |  |  |
| Bathing | 5 | 0 |  |  |  |
| Grooming (Washing face, brushing teeth, shaving, combing hair) | 5 | 0 |  |  |  |
| Dressing | 10 | 5 | 0 |  |  |
| Bowel control | 10 | 5（occasional incontinence） | 0（incontinence） |  |  |
| Urinary control | 10 | 5（occasional incontinence） | 0（incontinence） |  |  |
| Toileting (wiping, adjusting clothes, flushing) | 10 | 5 | 0 |  |  |
| Transferring | 15 | 10 | 5 | 0 |  |
| Walking 45 meters on level ground | 15 | 10 | 5 | 0 |  |
| Up and down stairs | 10 | 5 | 0 |  |  |

**Instructions for Filling Out the ADL (BI)**

The BI measures a patient's ten basic daily activities, such as eating, transferring, independently using the toilet, bathing, walking or dressing. Each item is divided into four levels of 0, 5, 10, 15 points according to the difficulty of the task, and the patient is evaluated accordingly. If the patient cannot complete the activity, the score of each item will be determined by the actual time and quantity of help needed. If the patient needs help, even if it is very little help or supervision, he cannot get full marks. When the patient cannot meet the specified standards, it is recorded as 0 points. If the patient gets the highest score (100 points), he should be able to control his bowel movements, eat by himself, get up or leave the chair, bathe independently, walk at least 45 meters, and be able to go up and down stairs. However, this only means that he can be alone, and does not mean that he can live independently (he may not be able to cook or clean the room).

Evaluation Guide

· This indicator should be used to record what the patient has done, not what the patient can do.

· Its main purpose is to determine the degree of independence of the patient in the absence of any help (whether verbal or physical, no matter how small or for what reason).

· In all the items tested, the patient is not independent when supervision is needed.

· The patient's performance should come from the most reliable evidence. It usually comes from the patient's friends, relatives and nurses, but direct observation and consensus are also important.

· Generally speaking, the patient's performance in the previous 24-48 hours is very important, but occasionally it is related to a longer time (for example, last week's bowel movement).

· Patients with unclear consciousness should be scored 0 points, even if there is no incontinence.

· Intermediate means that the patient's efforts exceed 50%.

· If you can control your urination yourself, the medium score means occasional urinary incontinence (less than or equal to 1 time/24 hours).

· Allow the use of assistive independent tools (such as crutches).

Use Guide

1. Eating: Independent eating means that the patient can independently eat prepared food within a normal time. The food includes any normal diet (not just porridge), and the food can be made or brought by others; picking up vegetables, serving rice, stirring, cutting food, etc. can be completed independently, scoring 10 points; if a small part of picking up vegetables, serving rice, stirring, cutting food, etc. need help to complete, score 5 points, otherwise score 0 points.

2. Bathing: No need for guidance, supervision and help can enter and exit the bathroom by themselves, wash themselves, shower does not need help or supervision, complete independently, score 5 points, otherwise score 0 points.

3. Grooming: refers to the situation within 24-48 hours, independently complete washing face, combing hair, brushing teeth, shaving and other personal hygiene, caregivers provide tools such as squeezing toothpaste, preparing water, etc, can also score 5 points, otherwise score 0 points.

4. Dressing: refers to being able to put on and take off various clothes, shoes and socks as before the illness, including personal ability to button, open and pull the zipper, wear shoes, etc, score 10 points; need others to help button, shoelaces, open and pull the zipper and other complex functions, but can independently put on outerwear, wear shoes and other simple functions score 5 points, otherwise score 0 points.

5. Bowel control: refers to the situation of a week; can fully control, score 10 points; occasionally (less than or equal to 1 time per week) incontinence, score 5 points; incontinence or coma more than 1 time per week is scored as 0 points.

6. Urinary control: refers to the situation within 24-48 hours; can fully control, score 10 points; occasionally (less than or equal to 1 time per 24 hours, more than 1 time per week urinary incontinence) incontinence, score 5 points; frequent urination (more than 1 time per 24 hours) incontinence, should be scored 0 points. Catheterized patients are classified as urinary incontinence.

7. Going to the toilet: can enter and exit the toilet or stool place by themselves, without others to undress or handle hygiene, score 10 points; if the above activities need help for some functions, score 5 points; if the main functions such as undressing and handling hygiene need help, score 0 points.

8. Chair/bed transfer: The patient can independently and safely move from the bed to the chair and return, score 15 points; to ensure safety, 1 person needs to help or language guidance, score 10 points; 2 people or 1 strong and skilled person need to help, score 5 points; cannot sit up, or need more than 2 people to help, score 0 points.

9. Walking (walking) on the ground: refers to the ability to use assistive tools (including crutches, but not rolling walking tools such as wheelchairs) to move in the home or ward or hospital, without supervision and care, can walk independently 45 meters, is considered to be completed independently, score 15 points; need 1 untrained person to help (physical or language guidance), including supervision and care, can walk 45 meters, score 10 points; can move independently on a wheelchair, independently use a wheelchair to walk 45 meters score 5 points, if not completed, score 0 points.

10. Up and down stairs: can independently go up and down stairs, including the use of assistive devices (such as crutches) to go up and down stairs, is still considered to be completed independently, score 10 points; under the partial help (such as support) or supervision of others, can complete up and down stairs, score 5 points, otherwise score 0 points.
